# Chitosan/Sodium Alginate/Velvet Antler Blood Peptides Hydrogel Promoted Wound Healing by Regulating PI3K/AKT/mTOR and SIRT1/NF-κB Pathways

**DOI:** 10.3389/fphar.2022.913408

**Published:** 2022-06-16

**Authors:** Mingqian Hao, Xiaojuan Peng, Shuwen Sun, Chuanbo Ding, Wencong Liu

**Affiliations:** ^1^ School of Chinese Medicinal Materials, Jilin Agricultural University, Changchun, China; ^2^ College of Traditional Chinese Medicine, Jilin Agricultural Science and Technology College, Jilin, China

**Keywords:** velvet antler blood peptides, hydrogel scaffold, skin wound, cell proliferation, angiogenesis, inflammation

## Abstract

Skin wound healing is a principal clinical challenge, and it is necessary to develop effective alternative treatments. Excessive inflammatory response is linked to delayed healing. This study was the first to report a multi-functional chitosan/sodium alginate/velvet antler blood peptides (VBPs) hydrogel (CAVBPH) and explore its potential mechanism to promote wound healing. The results showed that CAVBPH possessed desirable characteristics including thermo-sensitivity, antioxidation, antibacterial activity, biosafety, VBPs release behavior, etc., and significantly accelerated skin wound healing in mice. Specifically, the CAVBPH treatment enhanced cell proliferation, angiogenesis, and extracellular matrix (ECM) secretion, and also relieved inflammation at the wound site compared to the PBS-treated group and blank hydrogel scaffold-treated group. Mechanistically, the efficacy of CAVBPH might be related to the activation of the PI3K/AKT/mTOR and SIRT1/NF-κB pathways. Overall, CAVBPH seems to be a promising therapy for skin repair, probably relying on the abundant short-chain peptides in VBPs.

## Introduction

The skin is the largest tissue in the human body, which is essential for controlling body temperature, balancing fluids, and defending against exogenous pathogens (Chandika et al., 2015). Once a large area of the skin is damaged or removed, it will seriously affect human health, causing infection, amputation, and even death (Liang et al., 2019). A gauze is the most basic wound dressing in clinical practice, but it cannot effectively promote tissue repair due to the lack of biological activity, tissue adhesion, and other shortcomings. Moreover, conventional strategies such as autografts, allografts, and topical or systemic antibiotics are currently limited (Cao et al., 2020; Shafique et al., 2021). Therefore, the development of wound dressings with excellent functions is necessary.

At present, hydrogels have gradually become a novel option for wound dressings due to their desirable biochemical properties and skin-mimicking structure (Ghanbari et al., 2021). Jiang et al. found that VEGF mimicking peptide/gelatin methacrylate hydrogel with high porosity and water absorption could promote the proliferation of NIH3T3 cells and accelerate the healing of pig skin wounds by inducing CD31, α-SMA, Collagen I, and Collagen III expressions (Jang et al., 2021). Cheng et al. fabricated a K_2_(SL)_6_K_2_-bovine serum albumin hydrogel with sulfhydryl and silver ions as cross-linking agents, and demonstrated its role in promoting collagen deposition and angiogenesis in the early stages of wound healing (Cheng et al., 2020). Ouyang et al. prepared a chitosan (CS)–tilapia marine peptides hydrogel with antibacterial activity, and evaluated its effect on burn skin healing in rabbits (Ouyang et al., 2018). Besides, a recent study showed that catechol functionalized CS/β-glycerophosphate (β-GP)/oyster peptide microspheres hydrogel with favorable biosafety promoted skin wound healing in mice by inhibiting inflammatory factors and up-regulating Ki-67 and VEGF expressions (Zhang et al., 2021). The aforementioned studies confirm that hydrogel scaffolds loaded with bioactive proteins/peptides appear to be a promising alternative therapy for skin wounds. Regrettably, these reports are inadequate for the *in vitro* evaluation of hydrogels and an in-depth exploration of underlying mechanisms by which they promote wound healing.

Velvet antler blood (VB), the blood that remains in a velvet antler while being sawed, is a traditional Chinese medicine used for nourishing and restoring (Tseng et al., 2012). VB is rich in protein, which provides nutrients and a good environment for the growth and regeneration of velvet antler. Velvet antler blood peptides (VBPs), the main ingredients of VB, have been reported to have significant antioxidant, anti-fatigue, and immune-enhancing activities (Ma et al., 2009; Lv et al., 2021). Yet, it remains unknown whether VBPs are the key factors affecting tissue regeneration and further study is therefore required.

**SCHEME 1:**
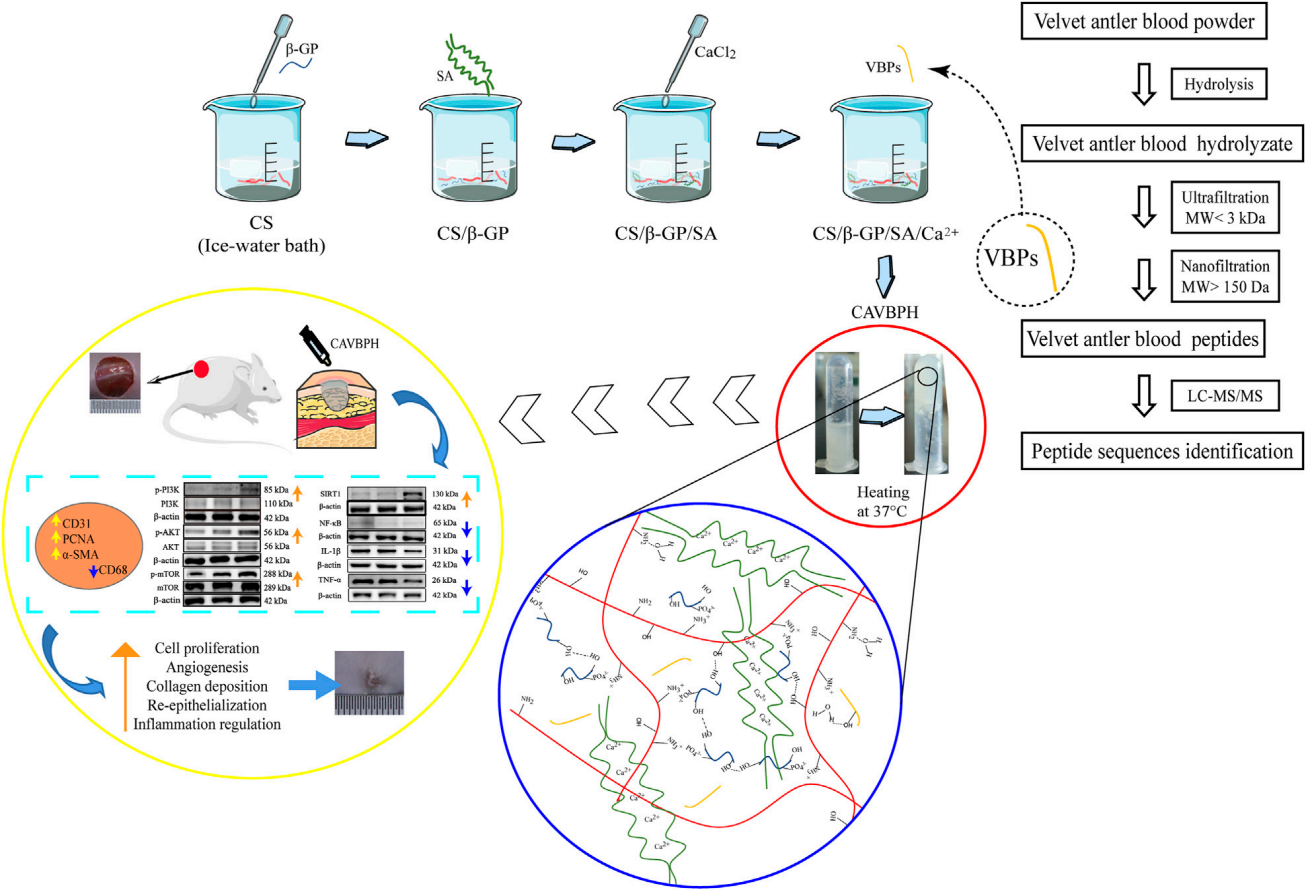
Schematic illustration of the synthesis procedure for CAVBPH and its application for accelerating skin wound healing.

Compared to traditional ointment vehicles (e.g., petrolatum) and other biomaterial hydrogels, the CS/sodium alginate (SA) hydrogel scaffold is more popular as wound dressing for topical drug delivery due to its thermosensitive, injectable, biocompatible, antibacterial, and tissue-regeneration properties (Ehterami et al., 2019; You et al., 2019; Zhang et al., 2021). Studies have found that the antioxidant capacity of animal-derived peptides with low molecular weights (MW) is likely to be related to the promotion of wound healing (Song et al., 2019; Yang et al., 2019). Thus, we speculate that the combination of CS/SA hydrogel scaffold with VBPs may be a promising dressing for skin wound healing. As shown in Scheme 1, VBPs prepared from VB were firstly introduced into the CS/SA scaffold cross-linked by β-GP and Ca^2+^ to fabricate a thermosensitive CS/SA/VBPs hydrogel (CAVBPH), and then, the *in vitro* characteristics of CAVBPH and its potential mechanism for affecting wound healing in mice were further explored. To the best of our knowledge, this is the first study to assess the biological effect of CAVBPH on wound healing.

## Materials and Methods

### Materials

VB powder came from Jinlu Pharmaceutical (Changchun, China). CS (from crab shells, deacetylation degree ≥95%, viscosity: 200–400 mPa s, MW: 160–400 kDa) and SA (from brown algae, MW: 32–250 kDa) were obtained from Meryer (Shanghai, China), and β-GP was from Yuanye (Shanghai, China). Pepsin (from porcine gastric mucosa, ≥ 250 u/mg) and l-glutathione (GSH) were purchased from Solarbio (Beijing, China), and pancreatin was from BIOFOUNT (Beijing, China). The human-immortalized keratinocytes (HaCat cells) were purchased from Cellcook Biotech (Guangzhou, China). *Escherichia coli* (*E. coil*), *Staphylococcus aureus* (*S. aureus*), and Luria–Bertani (LB) medium were obtained from Shanghai Luwei (Shanghai, China). Chloral hydrate was from Fuchen (Tianjin, China). The ultrafiltration membrane (MW: 3 kDa) and nanofiltration membrane (MW: 150 Da) were purchased from Jilin Hpewater (Jinlin, China). The RIPA lysis buffer (P0013B) and BCA protein assay kit (P0010) were from Beyotime (Shanghai, China). The primary antibody against CD31 (ab182981) came from Abcam (Cambridge, United Kingdom); PCNA (BM0104), α-SMA (BM0002), and CD68 (BA3638) were from BOSTER (Wuhan, China); p-PI3K (#4228) was from Cell Signaling Technology (Beverly, MA, United States); p-AKT (ARG51559) and p-mTOR (ARG40666) were acquired from Arigo (Hsinchu, Taiwan, China); and PI3K (67071-1-lg), AKT (60203-2-lg), mTOR (10745-1-AP), SIRT1 (13161-1-AP), NF-κB (p65, 10745-1-AP), IL-1β (26048-1-AP), TNF-α (17590-1-AP), β-actin (66009-1-lg), and horseradish peroxidase (HRP)–conjugated second antibodies (SA00001-1/SA00001-2) were purchased from Proteintech Group (Chicago, IL, United States). All other chemical agents were of analytical grade.

### Preparation of VBPs

VB was hydrolyzed by *in vitro* simulated gastrointestinal digestion (Zhao et al., 2019). Briefly, the VB solution (2%, w/v) was adjusted to pH 1.5 with 1 M HCl, and then pepsin (1 g/50 g VB) was added. The mixture was incubated at 37 °C, 120 rpm for 2 h to simulate gastric digestion. Subsequently, the pH of the solution was adjusted to 7.5 using 1 M NaOH, and pancreatin (1 g/25 g VB) was added to further digest for 4 h. The hydrolysate was heated at 95 °C for 15 min to inactivate the enzymes and then centrifuged at 8,000 rpm for 30 min. The supernatant was purified with an ultrafiltration membrane of 3 kDa and desalted using a 150 Da nanofiltration membrane to obtain VBPs. Subsequently, the VBPs solution was lyophilized (alpha 1–2 LD plus, Marin Christ, Germany) and stored at −20°C for further use.

### Composition Characteristics of VBPs

An ultimate 3,000 high-performance liquid chromatography system (HPLC, Thermo Fisher Scientific, United States) coupled with a Q Exactive™ Hybrid Quadrupole-Orbitrap™ mass spectrometer (Thermo Fisher Scientific, United States) with an ESI nanospray source and an amino acid analyzer (L-8900, Hitachi, Japan) were used to identify peptide sequences and the amino acid composition, respectively.

### Preparation of CAH and CAVBPH

CS (200 mg) was first gradually dissolved in 10 mL of 0.6% w/v acetic acid solution, and then a cold β-GP solution (55% w/v, 2.5 mL) was added dropwise to the cold CS solution with continuous stirring. After that, SA (100 mg) was added, and a CaCl_2_ solution (0.5% w/v, 0.2 mL) was dripped into the mixed solution under stirring to obtain CS/β-GP/SA/Ca^2+^ hydrogel (CAH). Then, VBPs were added into the CS/β-GP/SA/Ca^2+^ system to fabricate CAVBPH containing 1 mg/mL peptides. The sol-to-gel transition of the hydrogels at 37°C was measured by the centrifugal tube inverting method, which was based on the flow or no-flow standard of hydrogels when the centrifugal tube was inverted per minute at 37°C. 2 mL of CAH and CAVBPH both gelled within 2 min at 37°C, which indicated that the prepared hydrogels had a temperature-sensitive property. All solutions were freshly prepared and stored at 4 °C until use.

### Characterization of Hydrogels

The hydrogels were freeze-dried, and their microstructure was taken by using a scanning electron microscope (SEM; SS-550, Shimadzu, Japan). The characteristic functional groups of CS, β-GP, SA, VBPs, CAH, and CAVBPH were detected using a Fourier Transform Infrared (FTIR) spectrometer (FTIR-650, Tianjin Gangdong, China). The pH and viscosity of the hydrogels were, respectively, measured using a pH meter (PHS-3C, Shanghai Leici Instrument, China) and a viscometer with 3# rotor (NDJ-8S, Shanghai Precision & Scientific Instrument, China). The freeze-dried hydrogel was immersed in PBS at 37 °C for 48 h, and the swelling behavior of the hydrogels was analyzed as previously described (Zhang et al., 2020). The biodegradation profiles of CAH and CAVBPH within 14 days were plotted according to a previous report (Abueva and Lee, 2014). Subsequently, the release behavior of VBPs in CAVBPH was investigated. Briefly, CAVBPH in dryness (W_0_) was put into 20 mL of PBS under stirring (100 rpm). At different time points, 1 mL of the sample solution was collected and replaced with 1 mL of fresh PBS. The concentration of VBPs was determined using a BCA protein assay kit following the manufacturer’s instruction.

### Antioxidant and Hemolysis Tests

Hydrogels in dryness were immersed in PBS for 24 h to prepare sample solutions with different concentrations (1 mg/mL and 5 mg/mL), and VBPs and GSH solutions (1 mg/mL) were prepared as the positive control. The antioxidant activity was assessed by DPPH and ABTS radical scavenging methods as described previously (Xu C. et al., 2021). In the hemolytic assay, 2% mouse red blood cell (RBC) suspension (v/v, diluted with normal saline) was co-incubated with sample solutions of hydrogels or VBPs at 37 °C for 1 h. PBS-treated and 1% w/v TritonX-100–treated RBCs were used as negative and positive controls, respectively. The mixture was then centrifuged at 2,400 rpm for 10 min, and the absorbance of the supernatant was measured at 540 nm to calculate the hemolysis rate. Hemolysis rate (%) = (OD (sample)-OD (negative))/OD (positive) × 100%.

### Antibacterial Assay

The antibacterial activity was evaluated against *E. coil* (Gram-negative bacteria) and *S. aureus* (Gram-positive bacteria) according to the time-dependent co-culture test as mentioned previously with some modifications (Ghlissi et al., 2020; Karami et al., 2021). Briefly, all tested strains were cultured in an LB broth medium at 37°C to the logarithmic growth phase, and diluted to 10^5–6^ CFU/mL with normal saline. 0.5 mL of bacteria solution was mixed with 20 mL of the LB liquid medium containing 0.4 g UV-sterilized hydrogel and pre-incubated at 4°C for 1 h. No hydrogel samples were present in the control group. Then, all groups were incubated at 37°C and 100 rpm, and the absorbance of the LB medium in each group at 600 nm was measured regularly. After that, the co-culture solutions in all groups were appropriately diluted with an equal volume of normal saline, and further cultured on LB agar plates at 37°C for 12 h to observe the colony count.

### Cytotoxicity Evaluation

The cytotoxicity of the hydrogel on HaCaT cells was evaluated as previously described with minor modifications (Andrgie et al., 2020). Briefly, a UV-sterilized hydrogel was soaked in the culture medium at 37°C for 24 h to obtain the hydrogel medium. HaCaT cells were seeded in 96-well plates at a density of 0.5 × 10^4^ cells/well. After 24 h of cell adherence, the culture medium was replaced with the hydrogel medium, and incubated for another 24 h. The cytotoxicity was determined by comparing the survival rate of HaCaT cells in the presence and absence of hydrogels using the MTT assay.

### Skin Wound Model and Treatment

45 male ICR mice (18–20 g) were purchased from Changchun Yisi Experimental Animal Technology Co., Ltd. (Changchun, China), and were raised in a standard laboratory (22 ± 2°C, 60 ± 5% humidity and light alternated between day and night) with free access to food and water. After a week of adaptation, the ICR mice were anesthetized with chloral hydrate (400 mg/kg, i.p.), and a round full-thickness skin wound with a diameter of 1 cm was created after hair removal from the dorsal skin. Then, mice were randomly divided into three groups (*n* = 15/group), namely, the control group (treated with 200 μL of PBS), CAH group (treated with 200 μL of CAH), and CAVBPH group (treated with 200 μL of CAVBPH). On days 0, 3, 7, 10, and 14 after surgery, photographs of the wound areas were taken, and the wound size was measured using ImageJ software.

### Histological Analysis

Mice from each group were, respectively, sacrificed on days 7 (*n* = 7) and 14 (*n* = 8), and the tissue around the wound was harvested and fixed in 4% paraformaldehyde. Afterward, fixed tissues were embedded and sectioned for Hematoxylin and Eosin (H and E) staining, Masson’s trichrome (Masson) staining, and immunohistochemical (IHC) staining. IHC staining was performed with anti-CD31, anti-PCNA, anti-α-SMA, and anti-CD68 as described by Zhang et al. (Zhang et al., 2021). All stained sections were observed and photographed with a Nikon upright microscope equipped with a digital camera (Nikon Eclipse E100, Tokyo, Japan). Epidermal thickness and collagen content were quantified by ImageJ software, and the positive expression of four proteins was analyzed using Image-Pro Plus 6 software.

### Western Blotting

Skin wound tissues were homogenized in an ice-cold RIPA lysis buffer containing protease inhibitor, phosphatase inhibitor, and PMSF to extract total proteins. The protein concentrations of the samples in different groups were determined using a BCA kit, and 10% SDS-PAGE electrophoresis was performed. Then, the protein was transferred to the PVDF membrane and co-incubated with primary antibodies against PI3K, AKT, mTOR, p-PI3K, p-AKT, p-mTOR, SIRT1, NF-κB, IL-1β, TNF-α, and β-actin, as well as HRP-conjugated second antibodies for 2 and 1 h, respectively. The blots on the membranes were investigated by an enhanced chemiluminescent procedure and quantified with ImageJ software. β-actin was regarded as an internal loading control.

### Statistical Analysis

Data are expressed as mean ± standard deviation (SD). All experiments were carried out at least three times unless stated otherwise. IBM SPSS Statistics 23 software was used to analyze the results by one-way analysis of variance. *p* < 0.05 was considered statistically significant.

## Results

### The Composition of VBPs

The results of HPLC-MS/MS showed that VBPs had 372 short-chain peptide sequences (e.g., LKECCDKPV, FPH, LFP, EHF, and VGYP), 96.5% of which were not more than 10 amino acids in length (Supplementary Table S1). Notably, only a few identified peptides have been recorded with antioxidant, antibacterial, and anti-ACE activities in the BIOPEP-UWM database, but the biological activities of most sequences still need to be further investigated. Table 1 showed the hydrolyzed amino acids in VBPs, including eight essential amino acids (65.92%) and nine non-essential amino acids (34.08%). Lys (19.51%) was the most abundant amino acid, followed by Val (13.41%), Leu (13.25%), and Glu (10.15%). Hence, VBPs have low MW, good water solubility, and transdermal absorption capacity.

**TABLE 1:** Types and amino acid compositions of VBPs.

Amino Acid	Content (%)
Lys	19.51
Val	13.41
Leu	13.25
Glu	10.15
Phe	8.92
Ala	8.69
His	7.99
Asp	3.67
Cys	3.12
Gly	2.92
Pro	1.8
Tyr	1.73
Thr	1.5
Ser	1.4
Ile	1.12
Arg	0.59
Met	0.22

### Characterization of Hydrogels

**FIGURE 1:**
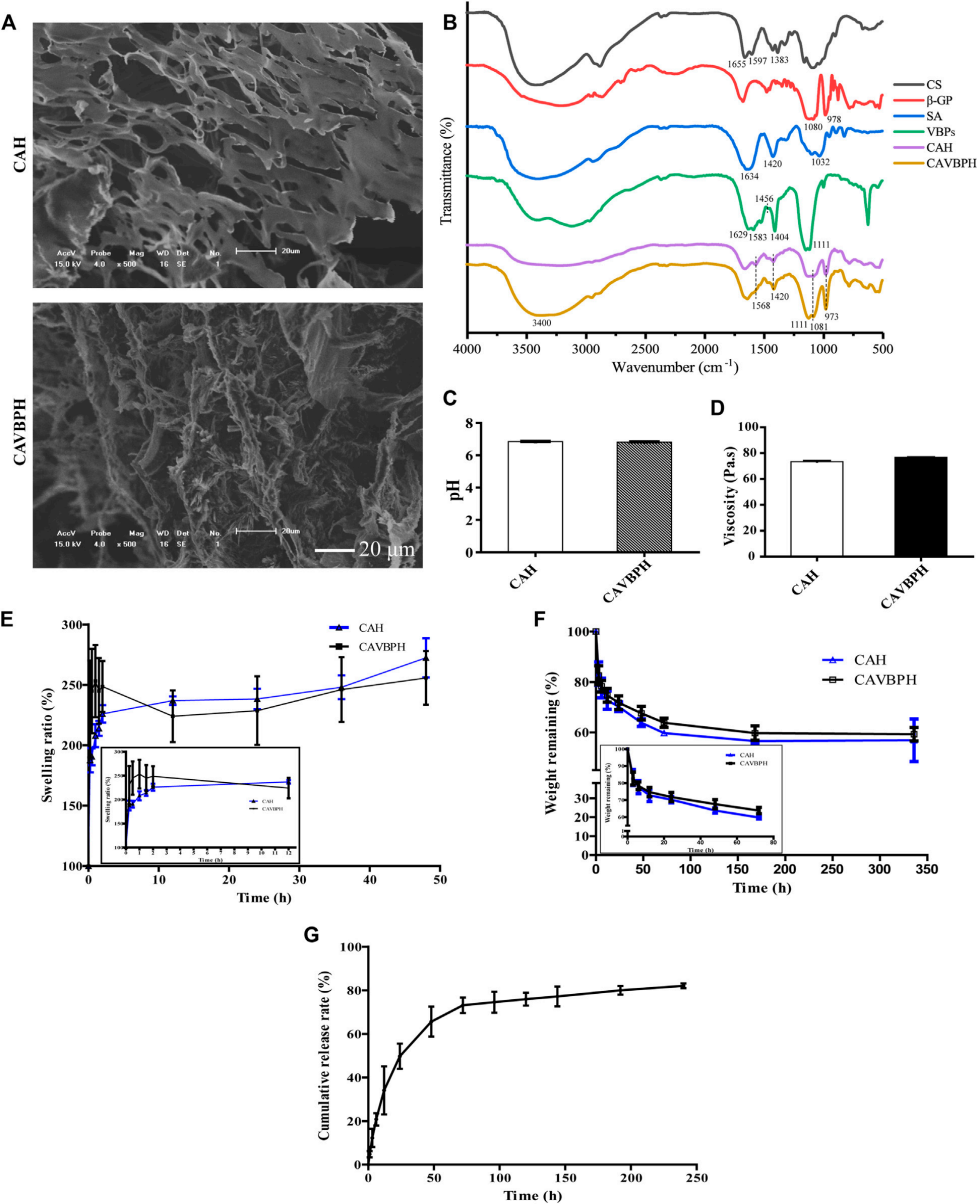
*In vitro* performance evaluation of hydrogels. **(A)** SEM micrograph (scale bar = 20 μm). **(B)** Infrared (IR) spectroscopy. **(C)** pH test. **(D)** Viscosity test. **(E)** Swelling test. **(F)** Biodegradation test. **(G)** *In vitro* release profile of VBPs.

The temperature-sensitive hydrogel was successfully fabricated as shown in Scheme 1. CAH and CAVBPH remained liquid at 25°C, and gelation occurred when the temperature rose to 37°C. The interaction forces of the CS/β-GP network mainly included the electrostatic effect and the intermolecular hydrogen bonds; moreover, the classic eggshell cross-linking structure between SA and Ca^2+^ further enhanced the mechanical properties of the hydrogel scaffold. Microscopically, both CAH and CAVBPH had a porous interconnected structure (Figure 1A), which not only facilitated moisture-locking but also supplied more oxygen to the wound bed. In addition, the porous structure of CAVBPH was also conducive to the release of VBPs.

The IR spectra of CS, β-GP, SA, VBPs, CAH, and CAVBPH are shown in Figure 1B. For CS, the absorption bands at 1,655 cm^−1^, 1,597 cm^−1^ , and 1,383 cm^−1^ represented amide I (C-O stretching), primary amine (N-H bending), and C-H bending vibration, respectively (Ouyang et al., 2018). For β-GP, the symmetric and asymmetric stretching vibration peaks of the phosphate ion (PO_4_^3-^) appeared near 978 cm^−1^ and 1,080 cm^−1^, respectively. The absorption band of the in-plane bending vibration of PO_4_^3-^ was at 800–500 cm^−1^ (Lisková et al., 2015; Zhang et al., 2021). In the IR spectrum of SA, the absorption peaks at 1,634 cm^−1^ and 1,420 cm^−1^ were, respectively, assigned to the symmetric stretching vibration and asymmetric stretching vibration of the carboxyl (C=O) bond, and the stretching vibration peak of C-O was located at 1,032 cm^−1^ (Wang et al., 2017). For CAH and CAVBPH, the symmetric and asymmetric stretching vibration peaks of PO_4_^3-^ (973 cm^−1^ and 1,081 cm^−1^) and the N-H vibration peak (1,568 cm^−1^) were weakened, indicating that there was an electrostatic interaction between -NH_3_^+^ and PO_4_^3-^. After cross-linking with Ca^2+^, the deformation vibration of O-H and the stretching vibration of C=O and C-O related to SA in the hydrogel scaffold were significantly weakened. In the IR spectrum of VBPs, the peaks at 1,629 cm^−1^ and 1,583 cm^−1^ were ascribed to the characteristic absorption of amides Ⅰ and Ⅱ, the bending vibrations of C-H and O-H were observed at 1,456 cm^−1^ and 1,404 cm^−1^, respectively, and the band of 1,111 cm^−1^ was caused by the stretching vibration of C-O (Ouyang et al., 2018; Zhang et al., 2021). CAVBPH exhibited the prominent characteristic peaks of VBPs at ∼3,400 cm^−1^, 1,583 cm^−1^, 1,404 cm^−1^ , and 1,111 cm^−1^, indicating that VBPs have been successfully encapsulated into the hydrogel scaffold.

The pH of the wound is generally in the weakly acidic or neutral range (5.4–7.4), which directly or indirectly plays an important role in wound healing. As shown in Figure 1C, the pH values of both CAH and CAVBPH were about 6.8. Moreover, the viscosities of CAH and CAVBPH were 73.49 Pa·s and 76.75 Pa·s (Figure 1D), respectively, which had stronger adhesion to tissues than several hydrogels reported recently (Tang et al., 2019; Zang et al., 2019; Yin et al., 2020). Good hygroscopicity corresponds to the capacity of dressing to absorb wound exudates. CAH and CAVBPH could quickly absorb water and reach a swelling ratio higher than 200% within the first 0.5 h, which was attributed to their highly porous structure and hydrophilic nature (Figure 1E). In addition, the weights of CAH and CAVBPH gradually decreased with the incubation time (Figure 1F). The rapid degradation occurred within the first 12 h. After incubation for 14 days, the weights of CAH and CAVBPH decreased from 100% to 56.85% and 59.26%, respectively. The slow degradation of the hydrogel indirectly reflected its long-time covering effect on the wound.

The porous structure of the hydrogel enables it to deliver bioactive molecules, cells, and drugs (Xu K. et al., 2021). The release behavior of VBPs from CAVBPH mainly included three processes: burst release, continuous release, and sustained release (Figure 1G). Within 12 h, it showed as a burst release with the release amount reaching 39.75%, which might be related to the high porosity of CAVBPH. Within 12–48 h, VBPs showed a nearly linear continuous release (72.53%). Then, the release process was still sustained with a slow speed and the cumulative release of VBPs reached 83.01% after 10 days, showing that CAVBPH could meet the requirements for VBP delivery.

### *In vitro* Biological Activity of Hydrogels

**FIGURE 2:**
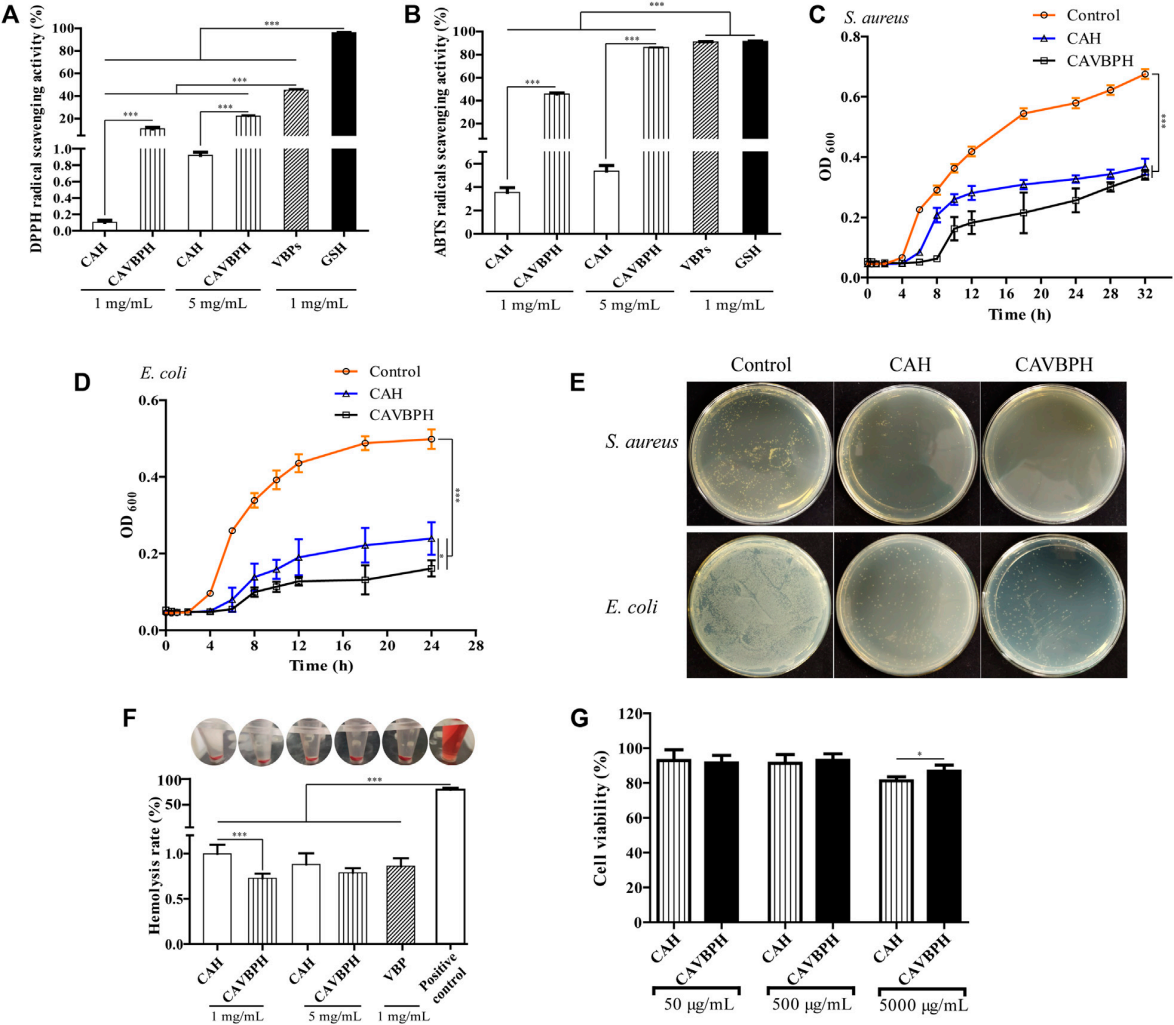
*In vitro* bioactivity analysis of hydrogels. **(A)** DPPH free-radical scavenging activity. **(B)** ABTS free-radical scavenging activity. **(C–E)** Bacterial growth and colony counting assays of hydrogels against *S. aureus* and *E. coli*. **(F)** Hemolysis analysis. **(G)** Cell viability of CAH and CAVBPH. *p < 0.05, **p < 0.01, ***p < 0.001.

In the present study, DPPH and ABTS free-radical scavenging activities of CAVBPH increased in a concentration-dependent manner and were significantly higher than the same concentrations of CAH at 1 mg/mL and 5 mg/mL (*p* < 0.001), while the free-radical scavenging abilities of VBPs and GSH were higher than those of CAVBPH and CAH (*p* < 0.001), which indicated that the antioxidant activity of CAVBPH was associated with the introduction of VBPs into the hydrogel scaffold (Figures 2A, B).

The antibacterial activities of CAH and CAVBPH against *S. aureus* (32 h) and *E. coli* (24 h) are shown in Figures 2C–E. Compared to the control group, both CAH and CAVBPH had a significant inhibitory effect on the growth and propagation of *S. aureus* and *E. coli* (*p* < 0.001), which might be related to the antibacterial nature of CS. In addition, the antibacterial effect of CAVBPH, especially against long-time *E. coli* (*p* < 0.05), was further improved due to the introduction of VBPs compared to CAH, thus probably reducing the chance of bacteria invading the wound.

The hemolysis rate of dressing less than 5% is considered to be clinically safe (Feng et al., 2016). In this study, the supernatants of the hydrogel-treated groups and VBPs-treated group tended to be colorless, but the positive control group was bright red (Figure 2F). Quantitative results showed that the hemolysis rates of CAH, CAVBPH, and VBPs were all less than 2% and hardly caused hemolysis, although there was a significant difference in the hemolysis rates between the CAH group and the CAVBPH group at the concentration of 1 mg/mL (*p* < 0.001). Overall, these results indicated that hydrogels and VBPs could hardly cause hemolysis *in vivo*.

As shown in Figure 2G, the vitality of HaCaT cells remained almost unchanged as the concentration of hydrogels increased from 50 μg/mL to 500 μg/mL. The cell viability of the CAVBPH group was significantly higher than that of CAH group at a concentration of 5,000 μg/mL (86.74 and 81.29%, respectively, *p* < 0.05). According to GB/T 16886.5-2003 (ISO 10993-5: 1999), the sample with a cell viability over 75% is generally considered non-cytotoxic (Yang et al., 2010). Thus, CAH and CAVBPH are considered to have good biosafety and negligible toxicity.

### Wound-Healing Efficiency

**FIGURE 3:**
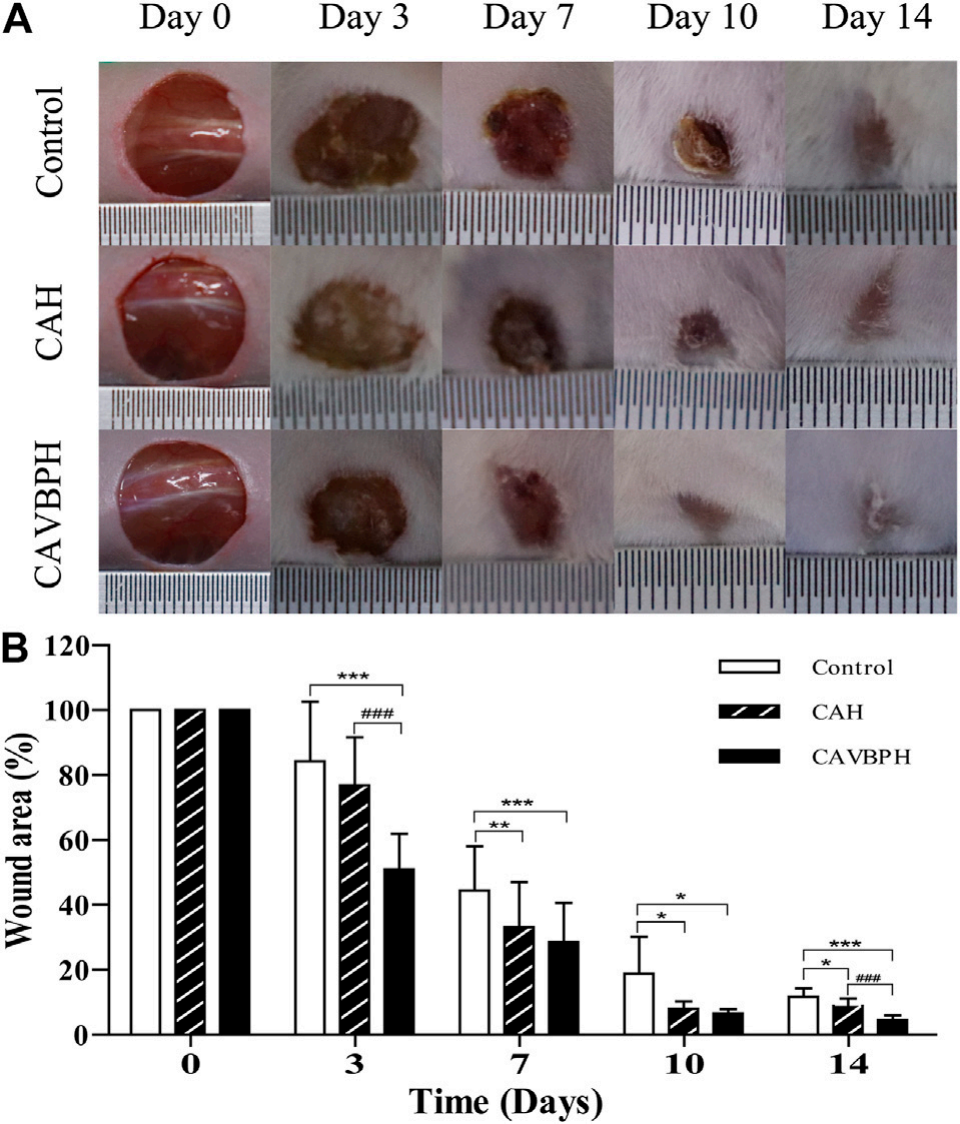
Macroscopic appearance analyses of skin wounds **(A)** and the wound area **(B)** in the different groups on days 0, 3, 7, 10, and 14. *p < 0.05, **p < 0.01, ***p < 0.001 *vs*. control group; #*p* < 0.05, ##*p* < 0.01, ###*p* < 0.001 *vs*. CAH group as statistically significant.

During the experiment, no obvious infections and other complications were observed in the wounds treated with CAH and CAVBPH, which further proved the biosafety of the prepared hydrogels. In addition, it could be seen that the wounds in all groups were gradually healing over time, but there were differences in their therapeutic performances (Figure 3). Compared to the control group, CAH treatment significantly shrank the area of the wound and accelerated the re-epithelialization on days 7, 10, and 14 (*p* < 0.05 or *p* < 0.01), which demonstrated that CAH was a promoting factor for wound healing. Notably, the CAVBPH group showed excellent wound shrinkage compared to the control group throughout the whole experimental period (*p* < 0.05 or *p* < 0.001) and significantly reduced the wound size particularly on days 3 and 14 compared to the control and CAH groups (*p* < 0.001), indicating that CAVBPH had a superior wound-healing property due to the sustained release of VBPs. The wounds in the CAVBPH group were generally healed without scabs on day 10 and even presented hair on day 14, while the CAH and control groups remained with unhealed small wounds. Collectively, these results showed that CAVBPH treatment significantly shortened wound-healing time.

### Histopathological Staining

**FIGURE 4:**
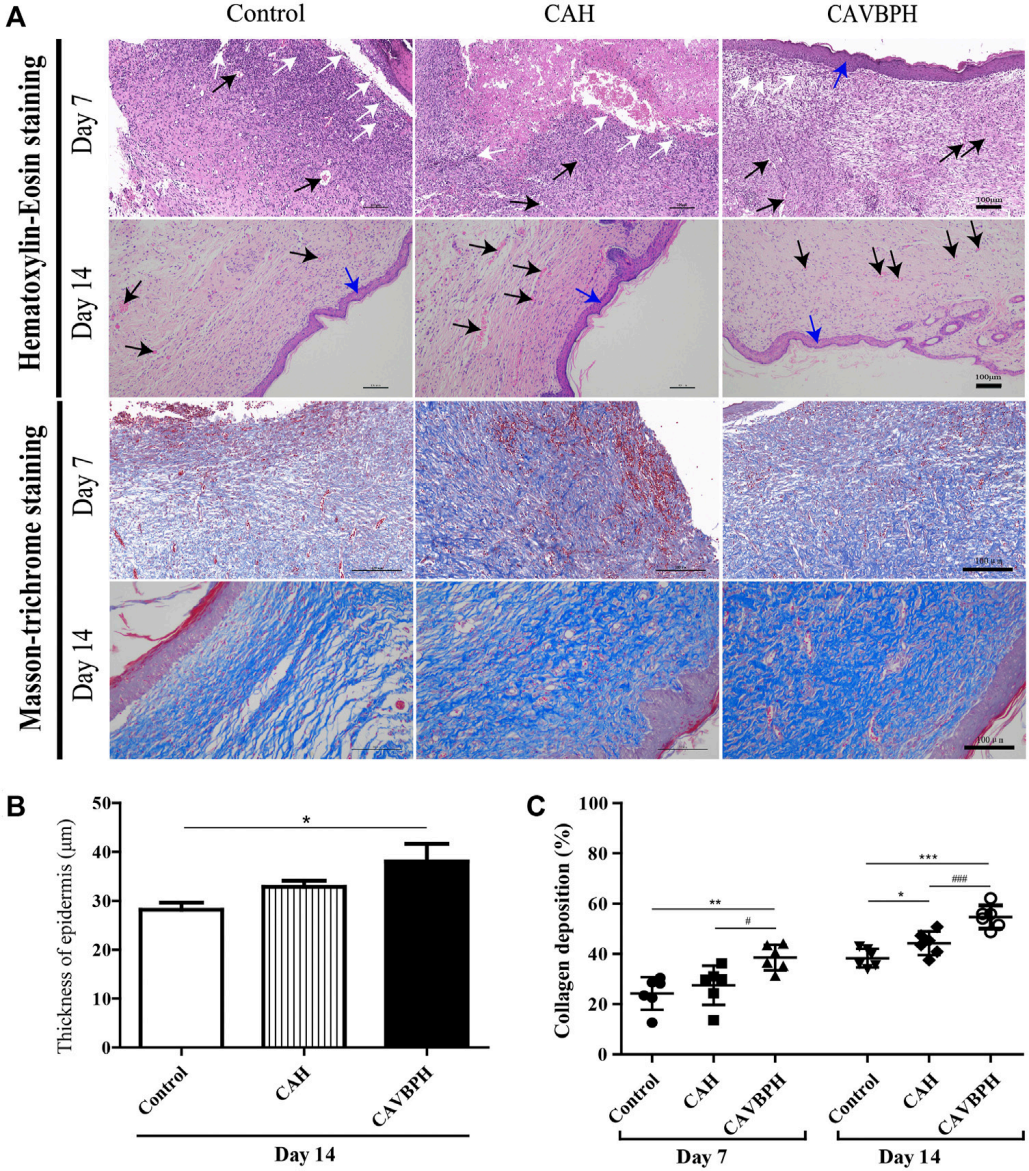
Histopathological analysis of skin wounds in the control, CAH, and CAVBPH groups’ mice on days 7 and 14. **(A)** H&E staining and Masson staining (scale bar = 100 μm). White arrows indicate inflammatory cells, black arrows indicate capillary vessels, and blue arrows indicate new epithelial tissues, respectively. **(B)** The thickness of the epithelial tissue on day 14. **(C)** Collagen content of skin wounds on days 7 and 14. *p < 0.05, **p < 0.01, ***p < 0.001 *vs*. control group; #*p* < 0.05, ##*p* < 0.01, ###*p* < 0.001 *vs*. CAH group as statistically significant.

As shown in Figures 4A, B, wound healing was evaluated by analyzing inflammatory cell infiltration, capillary formation, and epidermis thickness. On day 7, the control and CAH groups displayed more invasive lymphocytes (white arrows) than the CAVBPH group, suggesting an inflammatory response at the wound. The control and CAH groups lacked an epithelial layer, whereas the CAVBPH group showed an epidermis repair (blue arrows). In addition, the CAVBPH group had more capillaries (black arrows) than the other groups. On day 14, all groups showed relatively complete skin regeneration, fibroblast proliferation, and negligible inflammatory cells. Microscopically, the CAVBPH group possessed a more perfect fibroblast arrangement, capillary density, and epidermal structure than the CAH and control groups. Also, all groups exhibited increasing collagen fibers, but the CAVBPH group showed higher collagen content and more mature fiber structure than the CAH group and the control group on days 7 and 14 (*p <* 0.05, *p* < 0.01 or *p* < 0.001, Figures 4A, C).

### IHC Staining

**FIGURE 5:**
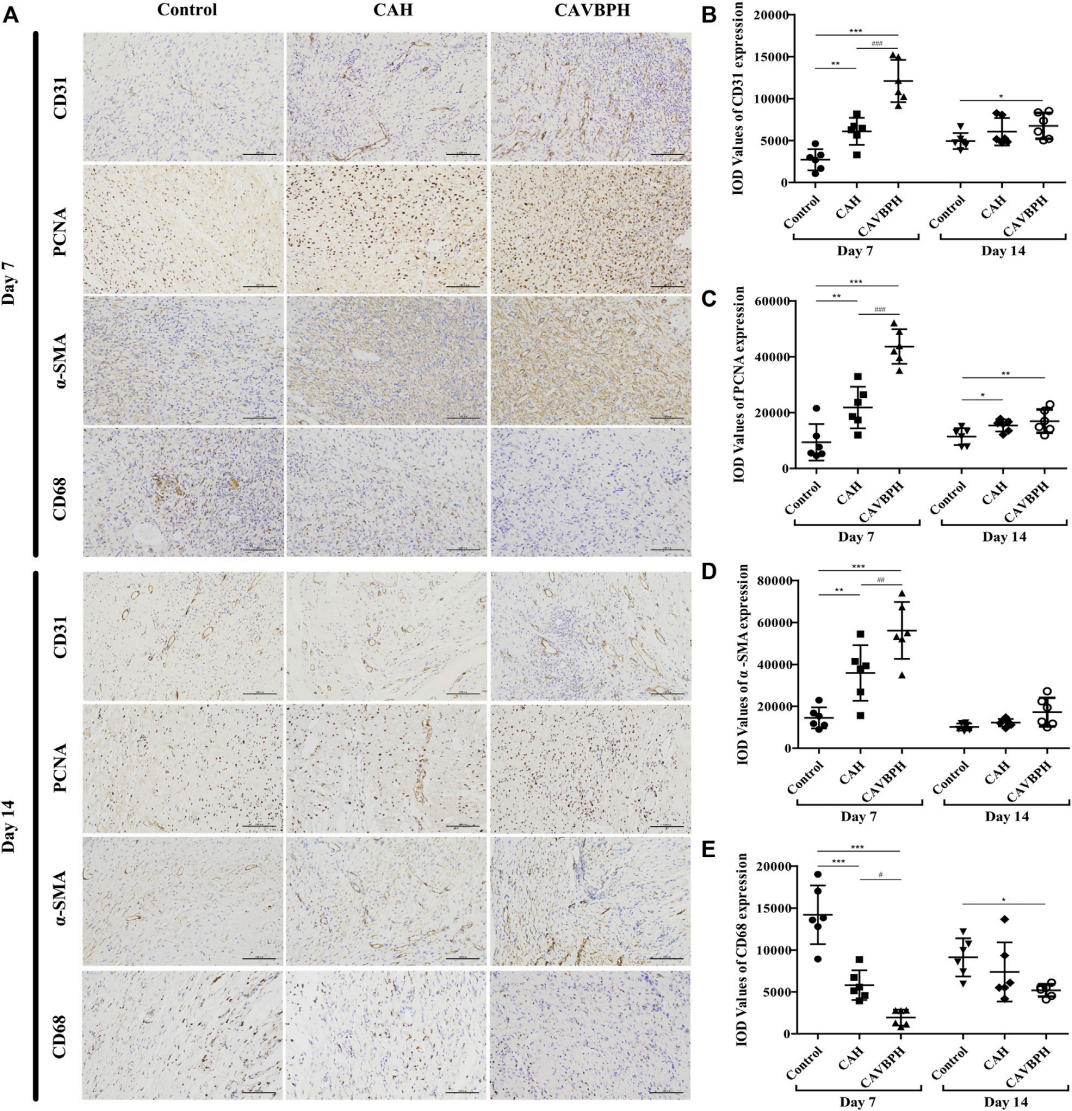
Immunohistochemistry staining of wound tissues on days 7 and 14. **(A)** Representative images for CD31, PCNA, α-SMA, and CD68 staining (scale bar = 100 μm). **(B–E)** Quantification of CD31, PCNA, α-SMA, and CD68 protein expressions, respectively. *p < 0.05, **p < 0.01, ***p < 0.001 *vs*. control group; #*p* < 0.05, ##*p* < 0.01, ###*p* < 0.001 *vs*. CAH group as statistically significant.

On day 7, the expression levels of CD31, PCNA, and α-SMA with hydrogel treatment were higher than those of the control group (*p* < 0.01 or *p* < 0.001, Figures 5A–D). Of note, CAVBPH treatment remarkably increased the expression of the aforementioned proteins when compared to the CAH group (*p* < 0.01 or *p* < 0.001). The control group showed the highest CD68 expression, while hydrogel treatment, especially CAVBPH intervention, significantly down-regulated the level of CD68 in wound tissues on day 7 (*p* < 0.001, Figures 5A, E). On day 14, the expression trends of the aforementioned four proteins in control, CAH, and CAVBPH groups were similar to those on day 7, indicating that CAVBPH achieved the desired effects on the regulation of CD31, PCNA, α-SMA, and CD68 proteins.

### Western Blotting

**FIGURE 6:**
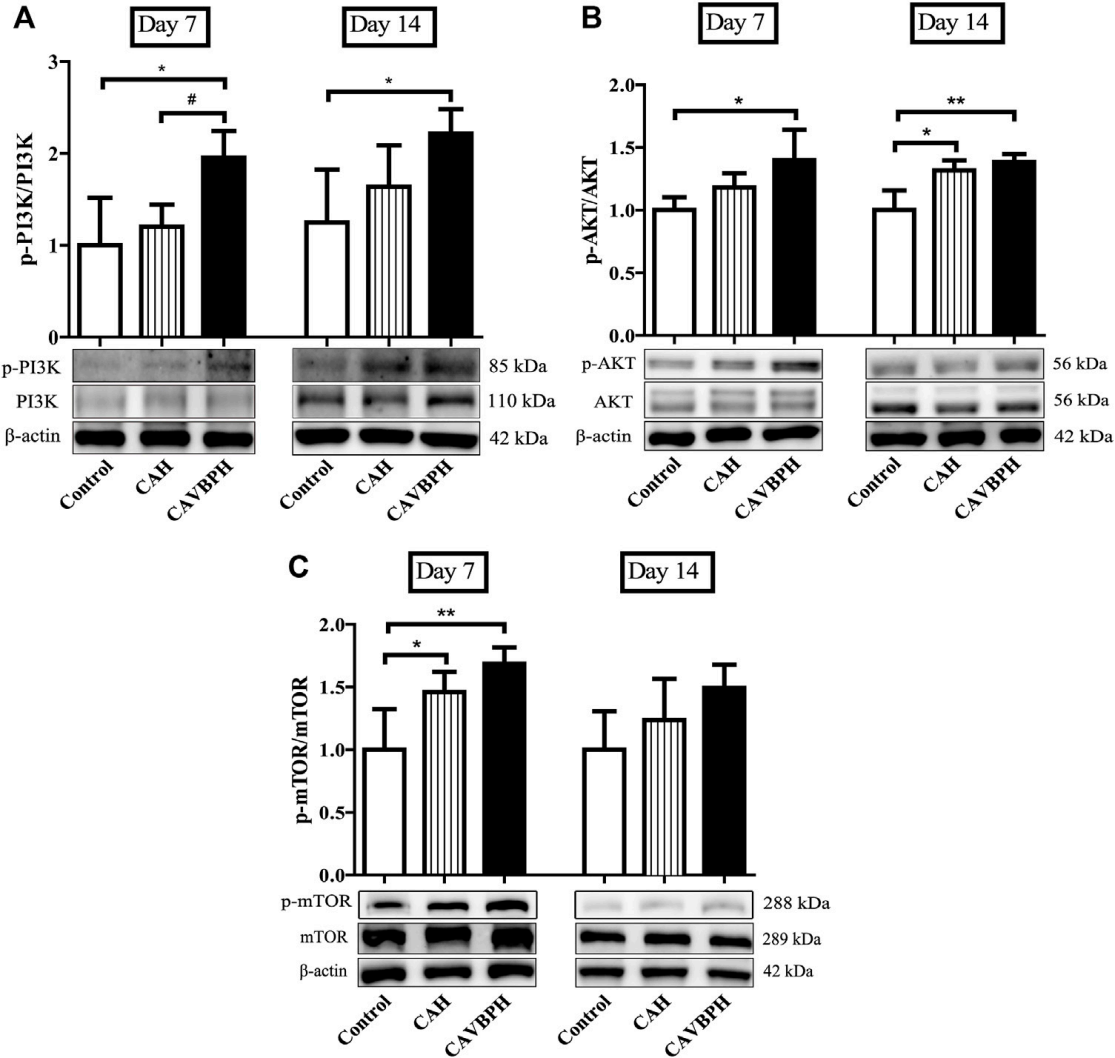
Western blotting analysis of the PI3K/AKT/mTOR pathway. **(A)** Protein bands and quantitative analyses of p-PI3K and PI3K. **(B)** Protein bands and quantitative analyses of p-AKT and AKT. **(C)** Protein bands and quantitative analyses of p-mTOR and mTOR. *p < 0.05, **p < 0.01, ***p < 0.001 *vs*. control; #*p* < 0.05, ##*p* < 0.01, ###*p* < 0.001 *vs*. CAH as statistically significant.

On day 7, CAVBPH treatment significantly up-regulated the phosphorylation levels of PI3K, AKT, and mTOR proteins in wound tissues compared to the control group (*p <* 0.05 or *p* < 0.01), and p-mTOR/mTOR was also increased in the CAH group (*p* < 0.05, Figure 6). On day 14, the expressions of p-PI3K/PI3K and p-AKT/AKT in the CAVBPH group were higher than those of the control group (*p* < 0.05 or *p* < 0.01, Figures 6A, B). In addition, CAH and CAVBPH both up-regulated the phosphorylation of p-mTOR/mTOR, although there was no significant difference compared to the control group (Figure 6C). Therefore, CAVBPH exhibited a stronger activation effect on the PI3K/AKT/mTOR pathway than CAH on days 7 and 14 of wound healing.

**FIGURE 7:**
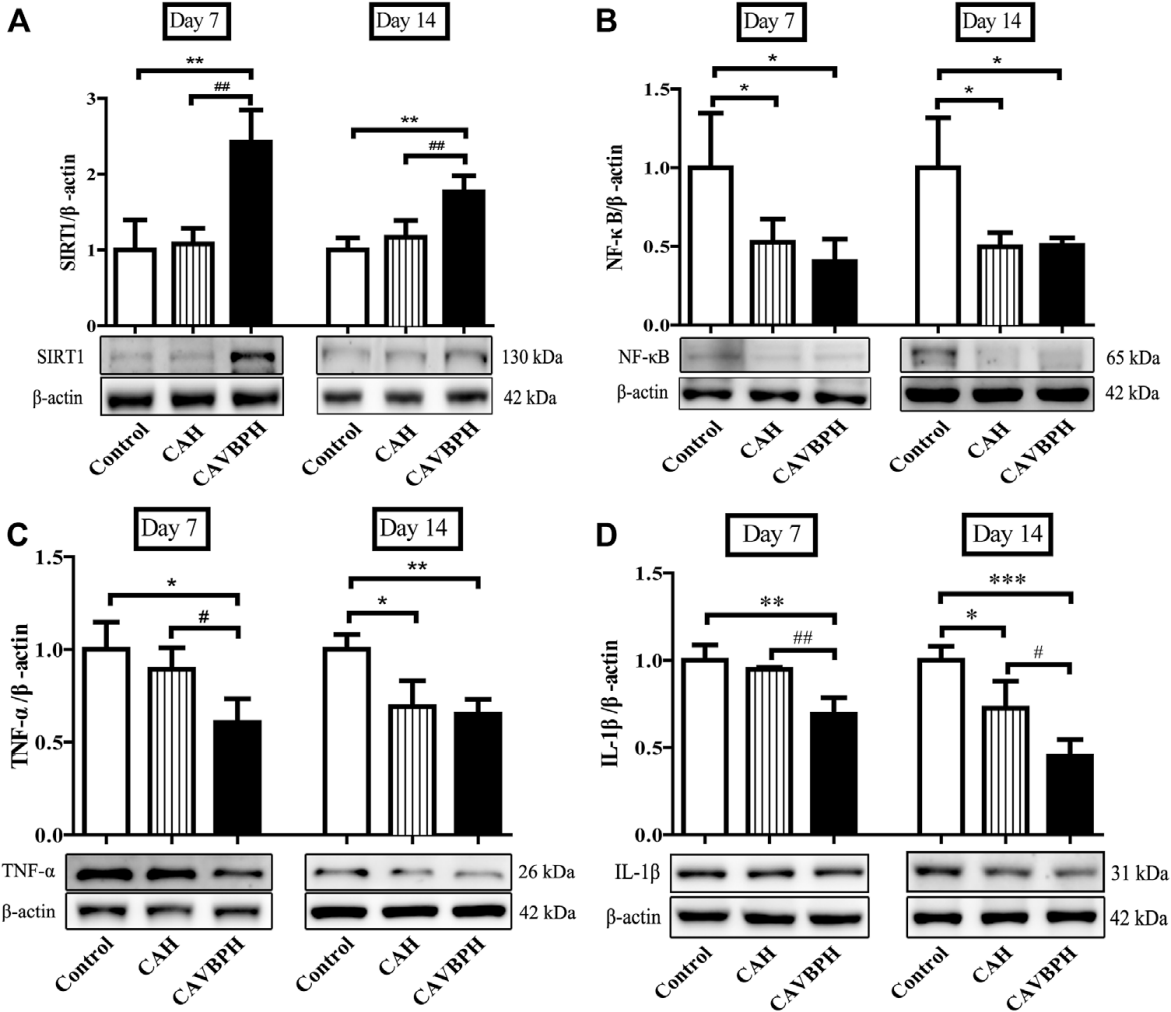
Western blotting analysis of proteins related to the SIRT1/NF-κB pathway. **(A)** Protein bands and quantitative analysis of SIRT1. **(B)** Protein bands and quantitative analysis of NF-κB. **(C)** Protein bands and quantitative analysis of TNF-α. **(D)** Protein bands and quantitative analysis of IL-1β. *p < 0.05, **p < 0.01, ***p < 0.001 *vs*. control group; #*p* < 0.05, ##*p* < 0.01, ###*p* < 0.001 *vs*. CAH group as statistically significant.

To demonstrate the anti-inflammatory effects of CAH and CAVBPH, we further assayed proteins involved in the SIRT1/NF-κB pathway (Figure 7). On day 7, SIRT1 was significantly up-regulated but NF-κB, TNF-α, and IL-1β were inhibited in the CAVBPH group compared to the control and CAH groups (*p <* 0.05 or *p* < 0.01). CAH intervention also up-regulated SIRT1 while down-regulating NF-κB, TNF-α, and IL-1β, although there was no significant difference compared to the control group. On day 14, CAH treatment down-regulated the expression of NF-κB, TNF-α, and IL-1β compared to the control group (*p* < 0.05). Also, the regulation effect of CAVBPH on the SIRT1/NF-κB pathway was significantly stronger than that of control group (*p <* 0.05, *p* < 0.01 or *p* < 0.001). Overall, CAVBPH could effectively regulate the expression of inflammatory proteins in wound tissues during skin repair.

## Discussion

Many extracts have been reported to be effective for wound healing, but the appropriate drug delivery systems are equally important in improving the therapeutic indices of active ingredients (Tasić-Kostov et al., 2019). Currently, hydrogels are regarded as a promising option for skin dressings and drug delivery, and the combined application of hydrophilic proteins/peptides and hydrogel scaffolds is gaining increasing attention in the treatment of skin wounds. Of note, animal-derived short-chain peptides normally exhibit excellent biological activities and could provide a nutritional matrix for the recovery and maintenance of damaged skin (Song et al., 2019; Yang et al., 2019). Therefore, we encapsulated short-chain VBPs with excellent antioxidant and low-hemolysis properties into a modern CS/SA hydrogel scaffold to investigate its skin repair ability *in vivo*.

In the characterization section, we confirmed the desirable parameters of CAH and CAVBPH, including thermo-sensitivity, porous structure, antibacterial activity, biosafety, and biocompatibility, which were conducive to carrying drugs, maintaining humidity balance and gas exchange in the wound, and defending against exogenous pathogens (Figure 1; Figure 2). Moreover, the addition of VBPs enhanced the antioxidant and antibacterial activities of CAVBPH compared to the blank hydrogel scaffold CAH. After skin injury, numerous aggressive free radicals are rapidly generated in the wound, which disrupt the local oxidative/antioxidant system, resulting in DNA fragmentation, lipid peroxidation, enzymatic inactivation, and delayed wound healing; conversely, dressings with antioxidant activity could improve oxidative stress and thus promote skin repair (Portugal et al., 2007). In addition, efficient antibacterial activity is one of the important properties of ideal dressings, which can protect wounds from bacterial invasion and proliferation and alleviate inflammatory responses (Yang et al., 2022). Cheng et al. demonstrated that the hydrogel loaded with cerium oxide nanoparticles and antimicrobial peptides possessed a strong free-radical scavenging ability and antibacterial activity, and accelerated wound repair (Cheng et al., 2021). Hamdi et al. found that blue crab chitosan/protein-composite hydrogels enriched with carotenoids had excellent antioxidant activity and promoted skin healing in rats (Hamdi et al., 2020). We confirmed that both CAH and CAVBPH could significantly inhibit the growth and proliferation of *S. aureus* and *E. coli*, and the antibacterial activity of CAVBPH was stronger compared to CAH (Figures 2C–E). The antibacterial effect of CAVBPH can be attributed to two reasons. The first is the natural antibacterial activity of CS, which interacts with negatively charged parts of the bacterial surface, resulting in bacterial death (Moeini et al., 2020). The second reason may be that some antimicrobial peptides in VBPs play a substantial role in bacterial growth (Schmitt et al., 2016). Therefore, we speculate that CAVBPH treatment can reduce the inflammatory response by scavenging excess free radicals and inhibiting bacterial invasion and growth at the wound site, ultimately contributing to rapid wound healing.

Zhao et al. found that CS-calcium alginate dressing had good antibacterial property with no cytotoxicity, and its ability to accelerate full-thickness skin wound healing in SD rats was better than calcium alginate dressing (Zhao et al., 2020). In this study, CAH and CAVBPH indeed accelerated the healing process of full-thickness skin defects (Figure 3). Notably, the wounds in the CAVBPH-treated group were basically healed on day 10 and covered with hair on day 14, exhibiting a superior wound healing–promoting effect than blank hydrogel CAH, recently reported hydrogels, and novel dihydroquercetin nanocomposite films (An et al., 2022; Mao et al., 2022; Zhang et al., 2022).

CD31 is a transmembrane protein expressed in early angiogenesis, which can reflect neovascularization during wound repair. PCNA protein is considered to be an important contributor to DNA replication during cell division and a marker of cell proliferation (Elmowafy et al., 2019), while the expression of α-SMA is recognized as an index of specific myofibroblasts and is related to the secretion of extracellular matrix (ECM) proteins (Elbialy et al., 2021). Moreover, the CD68 protein corresponds to M1 macrophages and can reflect the degree of inflammation at the wound (Yang et al., 2022). VEGF-mimicking peptide/gelatin methacrylate hydrogel has been proven to accelerate the healing of pig skin wounds by up-regulating CD31 and α-SMA proteins (Jang et al., 2021). Combined with the results of the H and E staining and Masson staining, CAVBPH treatment stimulated angiogenesis (capillaries and CD31^+^), cell proliferation (PCNA^+^), and collagen secretion (collagen fibers and α-SMA^+^) while relieving inflammation (CD68^−^), thus showing a macroscopic feature of promoting wound healing (Figure 4; Figure 5).

PI3K/AKT/mTOR is a classical signaling pathway that can affect wound healing by participating in different cellular processes such as proliferation, migration, differentiation, and survival (Bao et al., 2022). Zhang et al. found that notoginsenoside Ft1 promoted fibroblast proliferation by activating the PI3K/AKT/mTOR pathway and benefited wound healing in diabetic mice (Zhang et al., 2016). On days 7 and 14, CAVBPH might similarly activate cell proliferation in view of the higher phosphorylation levels of PI3K, AKT, and mTOR proteins compared to the CAH and control groups (Figure 6).

An abnormal expression of SIRT1 disrupts its downstream NF-κB signaling pathway, further leading to inflammatory dysregulation (Locksley et al., 2001). In addition, inflammatory reactions mediated by IL-1β and TNF-α could damage vascular endothelial cells and engender defective wound healing; conversely, blocking or silencing IL-1β or TNF-α inhibits macrophage infiltration and induces macrophage differentiation into healing-related phenotypes (Mirza et al., 2013; Kasiewicz and Whitehead, 2016). In the present study, hydrogel treatments, especially CAVBPH, up-regulated the level of SIRT1, while down-regulating the expressions of NF-κB, IL-1β, and TNF-α during the inflammatory phase and tissue-remodeling phase (Figure 7), implying that CAVBPH played a more effective role in preventing acute wound inflammation than blank hydrogel scaffold CAH by regulating the SIRT1/NF-κB pathway.

## Conclusion

This is the first study to use VBPs to enhance the biological properties of the CS/SA hydrogel scaffold. Taken together, according to the results of *in vitro* and *in vivo* experiments, we found that CAVBPH might be a promising therapy to repair skin wounds by alleviating inflammation and promoting cell proliferation and angiogenesis. The activation of PI3K/AKT/mTOR and SIRT1/NF-κB pathways is probably of great significance for improving the regenerative process of CAVBPH-treated wounds. The therapeutic effect of CAVBPH on skin injury is potentially associated with numerous short-chain peptides in VBPs, and further study is needed in the future.

## Data Availability

The original contributions presented in the study are included in the article/Supplementary Material; further inquiries can be directed to the corresponding authors.
