# Supplementary material for: Chitosan/Sodium Alginate/Velvet Antler Blood Peptides Hydrogel Promoted Wound Healing by Regulating PI3K/AKT/mTOR and SIRT1/NF-κB Pathways
Source: Front Pharmacol. 2022 Jun 16;13:913408. doi: 10.3389/fphar.2022.913408 (PMC9243309; doi:10.3389/fphar.2022.913408)
Supplement: Supplementary file 1 [file Table1.DOCX]

Supplementary Material

**Supplementary Table 1.** Identification of peptide sequences in VBPs.

| **Peptide** | **Observed m/z** | **z** | **Observed (M+H)** | **Starting position** | **Score** | **Protein Name** | **Scan Time** | **intensity** | **BIOPEP Activity** |
| --- | --- | --- | --- | --- | --- | --- | --- | --- | --- |
| TSTT | 409.188 | 1 | 409.188 | 496 | 42.8 | >tr\|F8V2T3\|F8V2T3_CERNI Tyrosine-protein kinase receptor OS=Cervus nippon OX=9863 PE=2 SV=1 | 29.0826 | 1998400000 |  |
| FWG | 409.187 | 1 | 409.187 | 13 | 154.2 | >tr\|A0A220IG97\|A0A220IG97_CERNI Adult beta-globin OS=Cervus nippon OX=9863 PE=3 SV=1 | 29.775 | 1998400000 |  |
| EHF | 432.188 | 1 | 432.188 | 42 | 243.3 | >tr\|A0A220IG97\|A0A220IG97_CERNI Adult beta-globin OS=Cervus nippon OX=9863 PE=3 SV=1 | 19.772 | 1540900000 |  |
| HHGGEFTPV | 490.735 | 2 | 980.463 | 116 | 443.3 | >tr\|A0A220IG97\|A0A220IG97_CERNI Adult beta-globin OS=Cervus nippon OX=9863 PE=3 SV=1 | 17.2045 | 756520000 |  |
| HHGGEFTP | 441.200 | 2 | 881.392 | 116 | 473.8 | >tr\|A0A220IG97\|A0A220IG97_CERNI Adult beta-globin OS=Cervus nippon OX=9863 PE=3 SV=1 | 12.5039 | 681960000 |  |
| MPGH | 441.179 | 1 | 441.179 | 432 | 4.9 | >tr\|A4GIN2\|A4GIN2_CERNI Collagen alpha-1(X) chain OS=Cervus nippon OX=9863 GN=COL10A1 PE=2 SV=1 | 30.5157 | 592870000 |  |
| FVP | 362.207 | 1 | 362.207 | 731 | 65.9 | >tr\|F8V2T3\|F8V2T3_CERNI Tyrosine-protein kinase receptor OS=Cervus nippon OX=9863 PE=2 SV=1 | 22.3382 | 484850000 | ACE inhibitor |
| PWT | 403.197 | 1 | 403.197 | 35 | 155.1 | >tr\|A0A220IG97\|A0A220IG97_CERNI Adult beta-globin OS=Cervus nippon OX=9863 PE=3 SV=1 | 15.3782 | 349250000 | antioxidative |
| LKECCDKPV | 574.777 | 2 | 1148.546 | 274 | 319.2 | >tr\|X2GM95\|X2GM95_CERNI Serum albumin (Fragment) OS=Cervus nippon OX=9863 PE=2 SV=1 | 10.8513 | 326360000 |  |
| ARHHGGEFTPV | 604.304 | 2 | 1207.600 | 114 | 449.1 | >tr\|A0A220IG97\|A0A220IG97_CERNI Adult beta-globin OS=Cervus nippon OX=9863 PE=3 SV=1 | 13.8694 | 235980000 |  |
| LFT | 380.217 | 1 | 380.217 | 505 | 73.5 | >tr\|X2GM95\|X2GM95_CERNI Serum albumin (Fragment) OS=Cervus nippon OX=9863 PE=2 SV=1 | 19.2577 | 189420000 |  |
| KFP | 391.235 | 1 | 391.235 | 221 | 192.9 | >tr\|X2GM95\|X2GM95_CERNI Serum albumin (Fragment) OS=Cervus nippon OX=9863 PE=2 SV=1 | 13.5351 | 188270000 |  |
| FKDLGEDNFQG | 635.291 | 2 | 1269.574 | 11 | 219.1 | >tr\|X2GM95\|X2GM95_CERNI Serum albumin (Fragment) OS=Cervus nippon OX=9863 PE=2 SV=1 | 32.5237 | 165910000 |  |
| KECCDKPVL | 383.519 | 3 | 1148.542 | 275 | 131.3 | >tr\|X2GM95\|X2GM95_CERNI Serum albumin (Fragment) OS=Cervus nippon OX=9863 PE=2 SV=1 | 10.8551 | 164540000 |  |
| PDPQVTVVAPGAEPESTQVQR | 1103.067 | 2 | 2205.127 | 32 | 33.7 | >tr\|D3YJ54\|D3YJ54_CERNI Insulin-like growth factor II OS=Cervus nippon OX=9863 GN=IGF2 PE=2 SV=1 | 61.4431 | 151140000 |  |
| FEHFGDL | 432.700 | 2 | 864.392 | 41 | 420.5 | >tr\|A0A220IG97\|A0A220IG97_CERNI Adult beta-globin OS=Cervus nippon OX=9863 PE=3 SV=1 | 39.6011 | 144210000 |  |
| TMPA | 435.189 | 1 | 435.189 | 825 | 74.5 | >tr\|F8V2T3\|F8V2T3_CERNI Tyrosine-protein kinase receptor OS=Cervus nippon OX=9863 PE=2 SV=1 | 13.9006 | 141300000 |  |
| AGLP | 357.221 | 1 | 357.221 | 136 | 1.7 | >tr\|F1APT7\|F1APT7_CERNI Toll-like receptor 8 OS=Cervus nippon OX=9863 GN=TLR8 PE=2 SV=1 | 14.2608 | 140940000 |  |
| DFQ | 409.177 | 1 | 409.177 | 128 | 63.2 | >tr\|A0A220IG97\|A0A220IG97_CERNI Adult beta-globin OS=Cervus nippon OX=9863 PE=3 SV=1 | 27.7937 | 136690000 |  |
| PLV | 328.223 | 1 | 328.223 | 425 | 85.4 | >tr\|F1APT7\|F1APT7_CERNI Toll-like receptor 8 OS=Cervus nippon OX=9863 GN=TLR8 PE=2 SV=1 | 16.7223 | 135380000 |  |
| PVL | 328.223 | 1 | 328.223 | 123 | 62.4 | >tr\|A0A220IG97\|A0A220IG97_CERNI Adult beta-globin OS=Cervus nippon OX=9863 PE=3 SV=1 | 17.2317 | 135380000 |  |
| TVYYKEAPFKNVTEYDGQDAC | 313.148 | 8 | 2498.130 | 524 | 62.1 | >tr\|F8V2T3\|F8V2T3_CERNI Tyrosine-protein kinase receptor OS=Cervus nippon OX=9863 PE=2 SV=1 | 22.4327 | 121300100 |  |
| HGGEFTPV | 422.204 | 2 | 843.401 | 117 | 443.0 | >tr\|A0A220IG97\|A0A220IG97_CERNI Adult beta-globin OS=Cervus nippon OX=9863 PE=3 SV=1 | 21.8217 | 117910000 |  |
| LSQKFPK | 424.256 | 2 | 847.505 | 218 | 426.1 | >tr\|X2GM95\|X2GM95_CERNI Serum albumin (Fragment) OS=Cervus nippon OX=9863 PE=2 SV=1 | 12.9615 | 114710000 |  |
| HPE | 382.187 | 1 | 382.187 | 337 | 135.4 | >tr\|X2GM95\|X2GM95_CERNI Serum albumin (Fragment) OS=Cervus nippon OX=9863 PE=2 SV=1 | 33.6888 | 108170000 |  |
| FSYHIHVKGTHAWVGLYKNGTPVMY | 969.174 | 3 | 2905.507 | 593 | 29.1 | >tr\|A4GIN2\|A4GIN2_CERNI Collagen alpha-1(X) chain OS=Cervus nippon OX=9863 GN=COL10A1 PE=2 SV=1 | 61.1458 | 100010000 |  |
| LLPV | 441.308 | 1 | 441.308 | 1173 | 100.4 | >tr\|F8V2T3\|F8V2T3_CERNI Tyrosine-protein kinase receptor OS=Cervus nippon OX=9863 PE=2 SV=1 | 32.1652 | 92923000 |  |
| AFW | 423.205 | 1 | 423.205 | 12 | 191.3 | >tr\|A0A220IG97\|A0A220IG97_CERNI Adult beta-globin OS=Cervus nippon OX=9863 PE=3 SV=1 | 42.1333 | 85373000 |  |
| TGKP | 402.222 | 1 | 402.222 | 105 | 79.7 | >tr\|A4GIN2\|A4GIN2_CERNI Collagen alpha-1(X) chain OS=Cervus nippon OX=9863 GN=COL10A1 PE=2 SV=1 | 28.3009 | 82013000 |  |
| VFR | 421.255 | 1 | 421.255 | 21 | 159.7 | >tr\|A0A2S1M4Y6\|A0A2S1M4Y6_CERNI Serum albumin OS=Cervus nippon OX=9863 PE=2 SV=1 | 15.5765 | 81868000 |  |
| FPH | 400.199 | 1 | 400.199 | 679 | 217.2 | >tr\|F1APT7\|F1APT7_CERNI Toll-like receptor 8 OS=Cervus nippon OX=9863 GN=TLR8 PE=2 SV=1 | 22.6714 | 80739000 |  |
| IAF | 350.207 | 1 | 350.207 | 25 | 216.4 | >tr\|X2GM95\|X2GM95_CERNI Serum albumin (Fragment) OS=Cervus nippon OX=9863 PE=2 SV=1 | 28.907 | 78061000 |  |
| LPV | 328.222 | 1 | 328.222 | 1174 | 155.5 | >tr\|F8V2T3\|F8V2T3_CERNI Tyrosine-protein kinase receptor OS=Cervus nippon OX=9863 PE=2 SV=1 | 15.3091 | 75332000 |  |
| GLLP | 399.261 | 1 | 399.261 | 1172 | 47.7 | >tr\|F8V2T3\|F8V2T3_CERNI Tyrosine-protein kinase receptor OS=Cervus nippon OX=9863 PE=2 SV=1 | 24.495 | 71028000 |  |
| HLVDEPQNLIK | 653.364 | 2 | 1305.720 | 378 | 402.7 | >tr\|X2GM95\|X2GM95_CERNI Serum albumin (Fragment) OS=Cervus nippon OX=9863 PE=2 SV=1 | 30.9914 | 68445000 |  |
| LPGAP | 454.269 | 1 | 454.269 | 292 | 86.6 | >tr\|A4GIN2\|A4GIN2_CERNI Collagen alpha-1(X) chain OS=Cervus nippon OX=9863 GN=COL10A1 PE=2 SV=1 | 24.8639 | 65521000 |  |
| ATHG | 385.187 | 1 | 385.187 | 252 | 90.5 | >tr\|D3YJ54\|D3YJ54_CERNI Insulin-like growth factor II OS=Cervus nippon OX=9863 GN=IGF2 PE=2 SV=1 | 26.4523 | 57964000 |  |
| IMIG | 449.241 | 1 | 449.241 | 58 | 99.8 | >tr\|A0A089G3A2\|A0A089G3A2_CERNI Cytochrome c oxidase subunit 1 (Fragment) OS=Cervus nippon OX=9863 GN=COI PE=3 SV=1 | 21.3832 | 55221000 |  |
| LSGNG | 447.226 | 1 | 447.226 | 910 | 44.7 | >tr\|F8V2T3\|F8V2T3_CERNI Tyrosine-protein kinase receptor OS=Cervus nippon OX=9863 PE=2 SV=1 | 21.3832 | 55221000 |  |
| EATC | 480.186 | 1 | 480.186 | 84 | 78.6 | >sp\|Q8WMR3\|GLHA_CERNI Glycoprotein hormones alpha chain OS=Cervus nippon OX=9863 GN=CGA PE=2 SV=1 | 20.7042 | 51895000 |  |
| LLP | 342.238 | 1 | 342.238 | 177 | 183.3 | >tr\|X2GM95\|X2GM95_CERNI Serum albumin (Fragment) OS=Cervus nippon OX=9863 PE=2 SV=1 | 20.0024 | 50894000 | ACE inhibitor |
| PVLAP | 496.316 | 1 | 496.316 | 1102 | 156.7 | >tr\|F8V2T3\|F8V2T3_CERNI Tyrosine-protein kinase receptor OS=Cervus nippon OX=9863 PE=2 SV=1 | 23.8556 | 49657000 |  |
| NGQV | 417.215 | 1 | 417.215 | 510 | 78.2 | >tr\|F1APT7\|F1APT7_CERNI Toll-like receptor 8 OS=Cervus nippon OX=9863 GN=TLR8 PE=2 SV=1 | 36.3406 | 47873000 |  |
| VDW | 419.195 | 1 | 419.195 | 155 | 122.7 | >tr\|F8V2T3\|F8V2T3_CERNI Tyrosine-protein kinase receptor OS=Cervus nippon OX=9863 PE=2 SV=1 | 27.9711 | 46201000 |  |
| SWL | 405.214 | 1 | 405.214 | 41 | 110.3 | >tr\|G9F9N5\|G9F9N5_CERNI Melatonin receptor OS=Cervus nippon OX=9863 GN=MTNR1A PE=3 SV=1 | 37.5061 | 45302000 |  |
| TLFP | 477.272 | 1 | 477.272 | 3 | 162.5 | >tr\|A0A158V0F9\|A0A158V0F9_CERNI Menin (Fragment) OS=Cervus nippon OX=9863 PE=2 SV=1 | 23.1365 | 43452000 |  |
| TNA | 305.157 | 1 | 305.157 | 606 | 14.6 | >tr\|F8V2T3\|F8V2T3_CERNI Tyrosine-protein kinase receptor OS=Cervus nippon OX=9863 PE=2 SV=1 | 17.3548 | 42707000 |  |
| TLF | 380.218 | 1 | 380.218 | 68 | 157.9 | >tr\|X2GM95\|X2GM95_CERNI Serum albumin (Fragment) OS=Cervus nippon OX=9863 PE=2 SV=1 | 31.188 | 41787000 |  |
| LSL | 332.217 | 1 | 332.217 | 23 | 164.5 | >tr\|F8V2T3\|F8V2T3_CERNI Tyrosine-protein kinase receptor OS=Cervus nippon OX=9863 PE=2 SV=1 | 27.086 | 40155000 |  |
| SKK | 362.242 | 1 | 362.242 | 931 | 7.5 | >tr\|F1APT7\|F1APT7_CERNI Toll-like receptor 8 OS=Cervus nippon OX=9863 GN=TLR8 PE=2 SV=1 | 23.5259 | 39448000 |  |
| LSF | 366.203 | 1 | 366.203 | 399 | 232.4 | >tr\|F8V2T3\|F8V2T3_CERNI Tyrosine-protein kinase receptor OS=Cervus nippon OX=9863 PE=2 SV=1 | 21.2847 | 39059000 |  |
| MLT | 380.186 | 1 | 380.186 | 1 | 162.4 | >tr\|A0A220IG97\|A0A220IG97_CERNI Adult beta-globin OS=Cervus nippon OX=9863 PE=3 SV=1 | 21.2118 | 37820000 |  |
| VGYP | 435.224 | 1 | 435.224 | 16 | 237.2 | >tr\|Q2TQ48\|Q2TQ48_CERNI MHC class II antigen (Fragment) OS=Cervus nippon OX=9863 GN=Ceni-DRB PE=4 SV=1 | 15.3595 | 35560000 |  |
| SAPF | 421.209 | 1 | 421.209 | 78 | 205.5 | >tr\|Q2TQ81\|Q2TQ81_CERNI MHC class II antigen (Fragment) OS=Cervus nippon OX=9863 GN=Ceni-DQB PE=4 SV=1 | 20.6937 | 33385000 |  |
| MAP | 318.145 | 1 | 318.145 | 285 | 96.4 | >tr\|G9F9N5\|G9F9N5_CERNI Melatonin receptor OS=Cervus nippon OX=9863 GN=MTNR1A PE=3 SV=1 | 29.3995 | 33265000 | ACE inhibitor |
| KQTALVELLK | 571.863 | 2 | 1142.719 | 524 | 428.1 | >tr\|X2GM95\|X2GM95_CERNI Serum albumin (Fragment) OS=Cervus nippon OX=9863 PE=2 SV=1 | 36.5153 | 30189000 |  |
| YICDNQDTLSSK | 722.325 | 2 | 1443.642 | 262 | 384.1 | >tr\|X2GM95\|X2GM95_CERNI Serum albumin (Fragment) OS=Cervus nippon OX=9863 PE=2 SV=1 | 19.4724 | 30153000 |  |
| TGLT | 391.235 | 1 | 391.235 | 34 | 95.4 | >tr\|V5LTF3\|V5LTF3_CERNI Cu/Zn superoxide dismutase (Fragment) OS=Cervus nippon OX=9863 PE=2 SV=1 | 26.9336 | 29465000 |  |
| CAAVDKEACF | 585.752 | 2 | 1170.498 | 558 | 361.1 | >tr\|X2GM95\|X2GM95_CERNI Serum albumin (Fragment) OS=Cervus nippon OX=9863 PE=2 SV=1 | 24.4715 | 28712000 |  |
| PSGH | 397.181 | 1 | 397.181 | 12 | 163.8 | >tr\|Q2TQB3\|Q2TQB3_CERNI MHC class II antigen (Fragment) OS=Cervus nippon OX=9863 GN=Ceni-DQA1 PE=4 SV=1 | 24.4526 | 28337000 |  |
| VPK | 343.234 | 1 | 343.234 | 497 | 138.8 | >tr\|X2GM95\|X2GM95_CERNI Serum albumin (Fragment) OS=Cervus nippon OX=9863 PE=2 SV=1 | 14.087 | 28315000 | ACE inhibitor |
| TETH | 487.219 | 1 | 487.219 | 225 | 169.3 | >tr\|F8V2T3\|F8V2T3_CERNI Tyrosine-protein kinase receptor OS=Cervus nippon OX=9863 PE=2 SV=1 | 18.1837 | 28247000 |  |
| KPP | 341.218 | 1 | 341.218 | 173 | 130.7 | >tr\|F8V2T3\|F8V2T3_CERNI Tyrosine-protein kinase receptor OS=Cervus nippon OX=9863 PE=2 SV=1 | 16.4899 | 28154000 |  |
| TPPH | 451.222 | 1 | 451.222 | 264 | 144.6 | >tr\|F8UQP8\|F8UQP8_CERNI Cytochrome b OS=Cervus nippon OX=9863 PE=3 SV=1 | 30.932 | 28005000 |  |
| VYP | 378.202 | 1 | 378.202 | 33 | 260.3 | >tr\|A0A220IG97\|A0A220IG97_CERNI Adult beta-globin OS=Cervus nippon OX=9863 PE=3 SV=1 | 21.6798 | 27885000 | ACE inhibitor |
| TYE | 412.187 | 1 | 412.187 | 929 | 78.4 | >tr\|F8V2T3\|F8V2T3_CERNI Tyrosine-protein kinase receptor OS=Cervus nippon OX=9863 PE=2 SV=1 | 33.9108 | 27579000 |  |
| TPIF | 477.274 | 1 | 477.274 | 150 | 89.7 | >tr\|G0Z3A2\|G0Z3A2_CERNI Catalase OS=Cervus nippon OX=9863 GN=CAT PE=2 SV=1 | 31.2671 | 27181000 |  |
| TLY | 396.213 | 1 | 396.213 | 797 | 149.0 | >tr\|F8V2T3\|F8V2T3_CERNI Tyrosine-protein kinase receptor OS=Cervus nippon OX=9863 PE=2 SV=1 | 18.7683 | 25397000 |  |
| LFP | 376.224 | 1 | 376.224 | 99 | 214.9 | >tr\|F8V2T3\|F8V2T3_CERNI Tyrosine-protein kinase receptor OS=Cervus nippon OX=9863 PE=2 SV=1 | 26.4656 | 25360000 |  |
| DLF | 394.198 | 1 | 394.198 | 392 | 174.1 | >tr\|X2GM95\|X2GM95_CERNI Serum albumin (Fragment) OS=Cervus nippon OX=9863 PE=2 SV=1 | 37.5766 | 23668000 |  |
| LGGNP | 457.227 | 1 | 457.227 | 761 | 120.8 | >tr\|F1APT7\|F1APT7_CERNI Toll-like receptor 8 OS=Cervus nippon OX=9863 GN=TLR8 PE=2 SV=1 | 26.3681 | 22627000 |  |
| VSAP | 373.219 | 1 | 373.219 | 77 | 147.2 | >tr\|Q2TQ81\|Q2TQ81_CERNI MHC class II antigen (Fragment) OS=Cervus nippon OX=9863 GN=Ceni-DQB PE=4 SV=1 | 8.7906 | 22523000 |  |
| TPP | 314.181 | 1 | 314.181 | 264 | 138.4 | >tr\|F8UQP8\|F8UQP8_CERNI Cytochrome b OS=Cervus nippon OX=9863 PE=3 SV=1 | 18.6793 | 22066000 |  |
| ARHHGGEFTP | 554.769 | 2 | 1108.532 | 114 | 387.8 | >tr\|A0A220IG97\|A0A220IG97_CERNI Adult beta-globin OS=Cervus nippon OX=9863 PE=3 SV=1 | 10.9146 | 21895000 |  |
| IPL | 342.239 | 1 | 342.239 | 613 | 50.9 | >tr\|F8V2T3\|F8V2T3_CERNI Tyrosine-protein kinase receptor OS=Cervus nippon OX=9863 PE=2 SV=1 | 26.4374 | 21786000 |  |
| TLP | 330.191 | 1 | 330.191 | 538 | 103.3 | >tr\|A0A2S1M4Y6\|A0A2S1M4Y6_CERNI Serum albumin OS=Cervus nippon OX=9863 PE=2 SV=1 | 23.0758 | 21253000 |  |
| VPD | 330.165 | 1 | 330.165 | 515 | 114.7 | >tr\|X2GM95\|X2GM95_CERNI Serum albumin (Fragment) OS=Cervus nippon OX=9863 PE=2 SV=1 | 23.0758 | 21253000 |  |
| TPK | 345.224 | 1 | 345.224 | 12 | 145.2 | >tr\|G9F9N5\|G9F9N5_CERNI Melatonin receptor OS=Cervus nippon OX=9863 GN=MTNR1A PE=3 SV=1 | 16.9245 | 20602000 |  |
| SNN | 334.140 | 1 | 334.140 | 165 | 68.9 | >tr\|F8V2T3\|F8V2T3_CERNI Tyrosine-protein kinase receptor OS=Cervus nippon OX=9863 PE=2 SV=1 | 23.8082 | 19988000 |  |
| VLI | 344.254 | 1 | 344.254 | 23 | 86.5 | >tr\|X2GM95\|X2GM95_CERNI Serum albumin (Fragment) OS=Cervus nippon OX=9863 PE=2 SV=1 | 26.6721 | 19179000 |  |
| LLY | 408.250 | 1 | 408.250 | 153 | 164.0 | >tr\|X2GM95\|X2GM95_CERNI Serum albumin (Fragment) OS=Cervus nippon OX=9863 PE=2 SV=1 | 28.2729 | 18943000 | immunostimulating |
| APEL | 429.236 | 1 | 429.236 | 150 | 125.7 | >tr\|X2GM95\|X2GM95_CERNI Serum albumin (Fragment) OS=Cervus nippon OX=9863 PE=2 SV=1 | 17.8713 | 18391000 |  |
| DVHP | 467.215 | 1 | 467.215 | 484 | 136.2 | >tr\|G0Z3A2\|G0Z3A2_CERNI Catalase OS=Cervus nippon OX=9863 GN=CAT PE=2 SV=1 | 12.6012 | 18216000 |  |
| PAGG | 301.140 | 1 | 301.140 | 207 | 63.7 | >tr\|A0A089G3A2\|A0A089G3A2_CERNI Cytochrome c oxidase subunit 1 (Fragment) OS=Cervus nippon OX=9863 GN=COI PE=3 SV=1 | 9.6126 | 18123000 |  |
| QGPK | 429.245 | 1 | 429.245 | 346 | 0.8 | >tr\|A4GIN2\|A4GIN2_CERNI Collagen alpha-1(X) chain OS=Cervus nippon OX=9863 GN=COL10A1 PE=2 SV=1 | 9.628 | 17512000 |  |
| NFL | 393.224 | 1 | 393.224 | 573 | 38.6 | >tr\|A0A2S1M4Y6\|A0A2S1M4Y6_CERNI Serum albumin OS=Cervus nippon OX=9863 PE=2 SV=1 | 10.2248 | 16625000 |  |
| EGF | 352.165 | 1 | 352.165 | 294 | 105.9 | >tr\|F8V2T3\|F8V2T3_CERNI Tyrosine-protein kinase receptor OS=Cervus nippon OX=9863 PE=2 SV=1 | 37.3265 | 16520000 |  |
| TLE | 362.207 | 1 | 362.207 | 355 | 134.7 | >tr\|X2GM95\|X2GM95_CERNI Serum albumin (Fragment) OS=Cervus nippon OX=9863 PE=2 SV=1 | 28.7275 | 16339000 |  |
| GDLF | 451.219 | 1 | 451.219 | 97 | 180.3 | >tr\|F8V2T3\|F8V2T3_CERNI Tyrosine-protein kinase receptor OS=Cervus nippon OX=9863 PE=2 SV=1 | 34.0491 | 15962000 |  |
| YVL | 394.234 | 1 | 394.234 | 421 | 94.3 | >tr\|F8V2T3\|F8V2T3_CERNI Tyrosine-protein kinase receptor OS=Cervus nippon OX=9863 PE=2 SV=1 | 27.7314 | 15003000 | antioxidative /antibacterial |
| TYN | 397.179 | 1 | 397.179 | 762 | 88.6 | >tr\|F8V2T3\|F8V2T3_CERNI Tyrosine-protein kinase receptor OS=Cervus nippon OX=9863 PE=2 SV=1 | 23.7194 | 14636000 |  |
| EYL | 424.210 | 1 | 424.210 | 83 | 113.9 | >tr\|F8V2T3\|F8V2T3_CERNI Tyrosine-protein kinase receptor OS=Cervus nippon OX=9863 PE=2 SV=1 | 25.7848 | 14559000 |  |
| KGGK | 389.255 | 1 | 389.255 | 1168 | 130.6 | >tr\|F8V2T3\|F8V2T3_CERNI Tyrosine-protein kinase receptor OS=Cervus nippon OX=9863 PE=2 SV=1 | 15.108 | 14530000 |  |
| WLH | 455.229 | 1 | 455.229 | 27 | 81.2 | >tr\|A0A090AZL1\|A0A090AZL1_CERNI Amelogenin, Y isoform (Fragment) OS=Cervus nippon OX=9863 GN=AMELY PE=3 SV=1 | 14.6416 | 14389000 |  |
| FAEDKEVCK | 375.846 | 3 | 1125.525 | 308 | 242.9 | >tr\|X2GM95\|X2GM95_CERNI Serum albumin (Fragment) OS=Cervus nippon OX=9863 PE=2 SV=1 | 11.9067 | 14140000 |  |
| PFT | 364.186 | 1 | 364.186 | 795 | 171.3 | >tr\|F8V2T3\|F8V2T3_CERNI Tyrosine-protein kinase receptor OS=Cervus nippon OX=9863 PE=2 SV=1 | 16.6676 | 14130000 |  |
| ECCHGDLL | 502.204 | 2 | 1003.401 | 243 | 311.8 | >tr\|X2GM95\|X2GM95_CERNI Serum albumin (Fragment) OS=Cervus nippon OX=9863 PE=2 SV=1 | 22.4821 | 14074000 |  |
| ECCDKPVLEK | 639.298 | 2 | 1277.588 | 300 | 412.2 | >tr\|A0A2S1M4Y6\|A0A2S1M4Y6_CERNI Serum albumin OS=Cervus nippon OX=9863 PE=2 SV=1 | 12.6371 | 13819000 |  |
| VGGLL | 458.299 | 1 | 458.299 | 948 | 194.9 | >tr\|F8V2T3\|F8V2T3_CERNI Tyrosine-protein kinase receptor OS=Cervus nippon OX=9863 PE=2 SV=1 | 31.0469 | 13089000 |  |
| YIHEFDGDEQ | 626.758 | 2 | 1252.509 | 16 | 107.9 | >tr\|Q2TQA0\|Q2TQA0_CERNI MHC class II antigen (Fragment) OS=Cervus nippon OX=9863 GN=Ceni-DQA2 PE=4 SV=1 | 22.0507 | 12767000 |  |
| KECCDKPV | 518.234 | 2 | 1035.460 | 275 | 299.0 | >tr\|X2GM95\|X2GM95_CERNI Serum albumin (Fragment) OS=Cervus nippon OX=9863 PE=2 SV=1 | 10.9174 | 12739000 |  |
| LWE | 447.227 | 1 | 447.227 | 1202 | 174.7 | >tr\|F8V2T3\|F8V2T3_CERNI Tyrosine-protein kinase receptor OS=Cervus nippon OX=9863 PE=2 SV=1 | 21.1849 | 12595000 | antioxidative |
| FSAL | 437.241 | 1 | 437.241 | 487 | 259.8 | >tr\|X2GM95\|X2GM95_CERNI Serum albumin (Fragment) OS=Cervus nippon OX=9863 PE=2 SV=1 | 27.8833 | 12139000 |  |
| LPPL | 439.293 | 1 | 439.293 | 301 | 97.9 | >tr\|X2GM95\|X2GM95_CERNI Serum albumin (Fragment) OS=Cervus nippon OX=9863 PE=2 SV=1 | 35.988 | 12091000 |  |
| RGFP | 476.264 | 1 | 476.264 | 170 | 32.4 | >tr\|A4GIN2\|A4GIN2_CERNI Collagen alpha-1(X) chain OS=Cervus nippon OX=9863 GN=COL10A1 PE=2 SV=1 | 38.017 | 11786000 |  |
| MVH | 402.168 | 1 | 402.168 | 194 | 70.5 | >tr\|F8UQP8\|F8UQP8_CERNI Cytochrome b OS=Cervus nippon OX=9863 PE=3 SV=1 | 20.9241 | 11771000 |  |
| SFY | 416.193 | 1 | 416.193 | 419 | 131.2 | >tr\|F8V2T3\|F8V2T3_CERNI Tyrosine-protein kinase receptor OS=Cervus nippon OX=9863 PE=2 SV=1 | 33.466 | 11022000 |  |
| TVMENFVAFVDK | 700.351 | 2 | 1399.694 | 545 | 242.0 | >tr\|X2GM95\|X2GM95_CERNI Serum albumin (Fragment) OS=Cervus nippon OX=9863 PE=2 SV=1 | 60.5848 | 10483000 |  |
| WGKVNVDVVG | 536.795 | 2 | 1072.582 | 14 | 179.2 | >tr\|A0A220IG97\|A0A220IG97_CERNI Adult beta-globin OS=Cervus nippon OX=9863 PE=3 SV=1 | 32.672 | 10168000 |  |
| CPF | 423.171 | 1 | 423.171 | 34 | 175.0 | >tr\|X2GM95\|X2GM95_CERNI Serum albumin (Fragment) OS=Cervus nippon OX=9863 PE=2 SV=1 | 38.1369 | 10129000 |  |
| VLAF | 449.277 | 1 | 449.277 | 94 | 166.9 | >tr\|D3YJ54\|D3YJ54_CERNI Insulin-like growth factor II OS=Cervus nippon OX=9863 GN=IGF2 PE=2 SV=1 | 36.172 | 10088000 |  |
| VSRQ | 489.286 | 1 | 489.286 | 880 | 57.0 | >tr\|F8V2T3\|F8V2T3_CERNI Tyrosine-protein kinase receptor OS=Cervus nippon OX=9863 PE=2 SV=1 | 25.901 | 9871800 |  |
| TLQH | 498.257 | 1 | 498.257 | 89 | 169.9 | >tr\|C9EEW2\|C9EEW2_CERNI ATPase (Fragment) OS=Cervus nippon OX=9863 GN=ATP7A PE=4 SV=1 | 16.0191 | 9867600 |  |
| SLW | 405.214 | 1 | 405.214 | 12 | 167.7 | >tr\|F8V2T3\|F8V2T3_CERNI Tyrosine-protein kinase receptor OS=Cervus nippon OX=9863 PE=2 SV=1 | 25.9587 | 9615800 |  |
| FIGV | 435.268 | 1 | 435.268 | 488 | 59.7 | >tr\|F1APT7\|F1APT7_CERNI Toll-like receptor 8 OS=Cervus nippon OX=9863 GN=TLR8 PE=2 SV=1 | 27.0659 | 9596500 |  |
| SMTP | 435.187 | 1 | 435.187 | 75 | 99.3 | >tr\|A0A090AZL1\|A0A090AZL1_CERNI Amelogenin, Y isoform (Fragment) OS=Cervus nippon OX=9863 GN=AMELY PE=3 SV=1 | 27.0659 | 9596500 |  |
| LLF | 392.254 | 1 | 392.254 | 16 | 118.3 | >tr\|F8V2T3\|F8V2T3_CERNI Tyrosine-protein kinase receptor OS=Cervus nippon OX=9863 PE=2 SV=1 | 40.4915 | 9453000 | ACE inhibitor |
| TFE | 396.181 | 1 | 396.181 | 747 | 150.6 | >tr\|F1APT7\|F1APT7_CERNI Toll-like receptor 8 OS=Cervus nippon OX=9863 GN=TLR8 PE=2 SV=1 | 21.2106 | 9371300 | antioxidative |
| CCDKPVLEK | 383.520 | 3 | 1148.545 | 301 | 133.2 | >tr\|A0A2S1M4Y6\|A0A2S1M4Y6_CERNI Serum albumin OS=Cervus nippon OX=9863 PE=2 SV=1 | 11.686 | 9017300 |  |
| TTHL | 471.246 | 1 | 471.246 | 41 | 91.3 | >sp\|Q9MZD5\|SUMO1_CERNI Small ubiquitin-related modifier 1 OS=Cervus nippon OX=9863 GN=SUMO1 PE=3 SV=1 | 16.4711 | 8848800 |  |
| FSVT | 453.227 | 1 | 453.227 | 828 | 54.5 | >tr\|F1APT7\|F1APT7_CERNI Toll-like receptor 8 OS=Cervus nippon OX=9863 GN=TLR8 PE=2 SV=1 | 19.9459 | 8795300 |  |
| DPH | 368.158 | 1 | 368.158 | 364 | 101.8 | >tr\|X2GM95\|X2GM95_CERNI Serum albumin (Fragment) OS=Cervus nippon OX=9863 PE=2 SV=1 | 20.2094 | 8747700 |  |
| SGNG | 334.139 | 1 | 334.139 | 911 | 48.6 | >tr\|F8V2T3\|F8V2T3_CERNI Tyrosine-protein kinase receptor OS=Cervus nippon OX=9863 PE=2 SV=1 | 34.2602 | 8716300 |  |
| TEF | 396.180 | 1 | 396.180 | 517 | 77.7 | >tr\|F1APT7\|F1APT7_CERNI Toll-like receptor 8 OS=Cervus nippon OX=9863 GN=TLR8 PE=2 SV=1 | 21.4092 | 8711100 |  |
| SPTS | 391.198 | 1 | 391.198 | 9 | 58.7 | >tr\|F8V2T3\|F8V2T3_CERNI Tyrosine-protein kinase receptor OS=Cervus nippon OX=9863 PE=2 SV=1 | 15.3567 | 8532300 |  |
| AHR | 383.203 | 1 | 383.203 | 141 | 47.4 | >tr\|A0A220IG97\|A0A220IG97_CERNI Adult beta-globin OS=Cervus nippon OX=9863 PE=3 SV=1 | 14.1477 | 7776300 |  |
| SGNP | 374.173 | 1 | 374.173 | 99 | 8.7 | >tr\|F1APT7\|F1APT7_CERNI Toll-like receptor 8 OS=Cervus nippon OX=9863 GN=TLR8 PE=2 SV=1 | 46.1624 | 7735600 |  |
| EFT | 396.167 | 1 | 396.167 | 120 | 14.1 | >tr\|A0A220IG97\|A0A220IG97_CERNI Adult beta-globin OS=Cervus nippon OX=9863 PE=3 SV=1 | 20.5769 | 7642500 |  |
| YVLP | 491.289 | 1 | 491.289 | 131 | 125.8 | >tr\|F8UQP8\|F8UQP8_CERNI Cytochrome b OS=Cervus nippon OX=9863 PE=3 SV=1 | 31.5285 | 7633200 |  |
| TEA | 320.160 | 1 | 320.160 | 705 | 54.6 | >tr\|F8V2T3\|F8V2T3_CERNI Tyrosine-protein kinase receptor OS=Cervus nippon OX=9863 PE=2 SV=1 | 17.2825 | 7602400 |  |
| YLL | 408.250 | 1 | 408.250 | 84 | 85.1 | >tr\|F8V2T3\|F8V2T3_CERNI Tyrosine-protein kinase receptor OS=Cervus nippon OX=9863 PE=2 SV=1 | 32.9434 | 7599500 | antioxidative |
| VGEY | 467.214 | 1 | 467.214 | 397 | 184.3 | >tr\|X2GM95\|X2GM95_CERNI Serum albumin (Fragment) OS=Cervus nippon OX=9863 PE=2 SV=1 | 12.4208 | 7301100 |  |
| VLAPP | 496.315 | 1 | 496.315 | 1103 | 135.9 | >tr\|F8V2T3\|F8V2T3_CERNI Tyrosine-protein kinase receptor OS=Cervus nippon OX=9863 PE=2 SV=1 | 22.6481 | 7177900 |  |
| FFE | 442.199 | 1 | 442.199 | 40 | 80.4 | >tr\|A0A220IG97\|A0A220IG97_CERNI Adult beta-globin OS=Cervus nippon OX=9863 PE=3 SV=1 | 45.2728 | 7032700 |  |
| LSE | 348.191 | 1 | 348.191 | 87 | 94.0 | >tr\|A0A220IG97\|A0A220IG97_CERNI Adult beta-globin OS=Cervus nippon OX=9863 PE=3 SV=1 | 40.2919 | 7003100 |  |
| VAE | 318.181 | 1 | 318.181 | 1143 | 59.7 | >tr\|F8V2T3\|F8V2T3_CERNI Tyrosine-protein kinase receptor OS=Cervus nippon OX=9863 PE=2 SV=1 | 15.9162 | 6954600 |  |
| PLIP | 439.294 | 1 | 439.294 | 309 | 158.6 | >tr\|G0Z3A2\|G0Z3A2_CERNI Catalase OS=Cervus nippon OX=9863 GN=CAT PE=2 SV=1 | 31.5011 | 6902100 |  |
| SSK | 321.173 | 1 | 321.173 | 271 | 42.3 | >tr\|X2GM95\|X2GM95_CERNI Serum albumin (Fragment) OS=Cervus nippon OX=9863 PE=2 SV=1 | 15.8881 | 6592300 |  |
| KYL | 423.263 | 1 | 423.263 | 136 | 102.4 | >tr\|X2GM95\|X2GM95_CERNI Serum albumin (Fragment) OS=Cervus nippon OX=9863 PE=2 SV=1 | 21.2706 | 6490000 |  |
| IGGF | 393.213 | 1 | 393.213 | 60 | 256.4 | >tr\|A0A089G3A2\|A0A089G3A2_CERNI Cytochrome c oxidase subunit 1 (Fragment) OS=Cervus nippon OX=9863 GN=COI PE=3 SV=1 | 33.1798 | 6392100 |  |
| APFP | 431.231 | 1 | 431.231 | 256 | 59.3 | >tr\|F1APT7\|F1APT7_CERNI Toll-like receptor 8 OS=Cervus nippon OX=9863 GN=TLR8 PE=2 SV=1 | 37.5895 | 6239800 |  |
| ILG | 302.208 | 1 | 302.208 | 408 | 57.3 | >tr\|F8V2T3\|F8V2T3_CERNI Tyrosine-protein kinase receptor OS=Cervus nippon OX=9863 PE=2 SV=1 | 26.2768 | 6123700 |  |
| LFE | 408.214 | 1 | 408.214 | 393 | 182.7 | >tr\|X2GM95\|X2GM95_CERNI Serum albumin (Fragment) OS=Cervus nippon OX=9863 PE=2 SV=1 | 17.2237 | 5940100 |  |
| IFK | 407.266 | 1 | 407.266 | 355 | 137.0 | >tr\|F8V2T3\|F8V2T3_CERNI Tyrosine-protein kinase receptor OS=Cervus nippon OX=9863 PE=2 SV=1 | 17.2237 | 5940100 |  |
| TADAV | 476.230 | 1 | 476.230 | 49 | 37.4 | >tr\|A0A220IG97\|A0A220IG97_CERNI Adult beta-globin OS=Cervus nippon OX=9863 PE=3 SV=1 | 16.8343 | 5803000 |  |
| SLLEYFPH | 335.843 | 3 | 1005.514 | 674 | 166.4 | >tr\|F1APT7\|F1APT7_CERNI Toll-like receptor 8 OS=Cervus nippon OX=9863 GN=TLR8 PE=2 SV=1 | 18.4886 | 5754500 |  |
| ARH | 383.203 | 1 | 383.203 | 114 | 6.1 | >tr\|A0A220IG97\|A0A220IG97_CERNI Adult beta-globin OS=Cervus nippon OX=9863 PE=3 SV=1 | 9.7634 | 5658700 |  |
| LMP | 360.192 | 1 | 360.192 | 303 | 34.5 | >tr\|F8UQP8\|F8UQP8_CERNI Cytochrome b OS=Cervus nippon OX=9863 PE=3 SV=1 | 35.5358 | 5536100 |  |
| CCHGDLLECA | 617.739 | 2 | 1234.470 | 244 | 130.5 | >tr\|X2GM95\|X2GM95_CERNI Serum albumin (Fragment) OS=Cervus nippon OX=9863 PE=2 SV=1 | 28.3187 | 5494200 |  |
| TVL | 332.218 | 1 | 332.218 | 569 | 156.9 | >tr\|A0A2S1M4Y6\|A0A2S1M4Y6_CERNI Serum albumin OS=Cervus nippon OX=9863 PE=2 SV=1 | 18.0788 | 5430100 |  |
| SFGM | 441.177 | 1 | 441.177 | 1009 | 104.9 | >tr\|F8V2T3\|F8V2T3_CERNI Tyrosine-protein kinase receptor OS=Cervus nippon OX=9863 PE=2 SV=1 | 34.0619 | 5339900 |  |
| TQRFFEH | 482.738 | 2 | 964.468 | 37 | 407.5 | >tr\|A0A220IG97\|A0A220IG97_CERNI Adult beta-globin OS=Cervus nippon OX=9863 PE=3 SV=1 | 18.4325 | 5339300 |  |
| SYY | 432.188 | 1 | 432.188 | 642 | 27.3 | >tr\|F8V2T3\|F8V2T3_CERNI Tyrosine-protein kinase receptor OS=Cervus nippon OX=9863 PE=2 SV=1 | 15.1056 | 5321000 | antioxidative |
| FGDL | 451.219 | 1 | 451.219 | 44 | 289.4 | >tr\|A0A220IG97\|A0A220IG97_CERNI Adult beta-globin OS=Cervus nippon OX=9863 PE=3 SV=1 | 28.734 | 5298800 |  |
| GGAP | 301.141 | 1 | 301.141 | 399 | 40.6 | >tr\|G0Z3A2\|G0Z3A2_CERNI Catalase OS=Cervus nippon OX=9863 GN=CAT PE=2 SV=1 | 23.6153 | 5287600 |  |
| VSAF | 423.236 | 1 | 423.236 | 545 | 152.4 | >tr\|A4GIN2\|A4GIN2_CERNI Collagen alpha-1(X) chain OS=Cervus nippon OX=9863 GN=COL10A1 PE=2 SV=1 | 14.6981 | 5252600 |  |
| NVFP | 476.252 | 1 | 476.252 | 4 | 100.2 | >tr\|A0A142EHU4\|A0A142EHU4_CERNI Matrix metallopeptidase (Fragment) OS=Cervus nippon OX=9863 GN=MMP-13 PE=2 SV=1 | 30.3333 | 5243100 |  |
| EACFAVEGPK | 554.266 | 2 | 1107.525 | 564 | 383.9 | >tr\|X2GM95\|X2GM95_CERNI Serum albumin (Fragment) OS=Cervus nippon OX=9863 PE=2 SV=1 | 25.5984 | 5204200 |  |
| LPF | 376.223 | 1 | 376.223 | 185 | 180.4 | >tr\|F8UQP8\|F8UQP8_CERNI Cytochrome b OS=Cervus nippon OX=9863 PE=3 SV=1 | 32.4674 | 5112800 | ACE inhibitor |
| SFGM | 457.173 | 1 | 457.173 | 1009 | 81.0 | >tr\|F8V2T3\|F8V2T3_CERNI Tyrosine-protein kinase receptor OS=Cervus nippon OX=9863 PE=2 SV=1 | 25.9738 | 4942000 |  |
| SLHTLFGDELCK | 473.906 | 3 | 1419.703 | 65 | 494.7 | >tr\|X2GM95\|X2GM95_CERNI Serum albumin (Fragment) OS=Cervus nippon OX=9863 PE=2 SV=1 | 42.2616 | 4785000 |  |
| ETYGDMADCCEK | 739.765 | 2 | 1478.523 | 82 | 128.6 | >tr\|X2GM95\|X2GM95_CERNI Serum albumin (Fragment) OS=Cervus nippon OX=9863 PE=2 SV=1 | 21.6019 | 4751900 |  |
| EVSF | 481.232 | 1 | 481.232 | 1276 | 165.5 | >tr\|F8V2T3\|F8V2T3_CERNI Tyrosine-protein kinase receptor OS=Cervus nippon OX=9863 PE=2 SV=1 | 23.9006 | 4547300 |  |
| TPT | 318.166 | 1 | 318.166 | 419 | 100.7 | >tr\|X2GM95\|X2GM95_CERNI Serum albumin (Fragment) OS=Cervus nippon OX=9863 PE=2 SV=1 | 22.6896 | 4493300 |  |
| TVM | 366.179 | 1 | 366.179 | 545 | 38.3 | >tr\|X2GM95\|X2GM95_CERNI Serum albumin (Fragment) OS=Cervus nippon OX=9863 PE=2 SV=1 | 24.4568 | 4407800 |  |
| IALP | 413.278 | 1 | 413.278 | 938 | 60.4 | >tr\|F8V2T3\|F8V2T3_CERNI Tyrosine-protein kinase receptor OS=Cervus nippon OX=9863 PE=2 SV=1 | 31.8176 | 4259900 |  |
| FGHP | 457.221 | 1 | 457.221 | 223 | 219.7 | >tr\|A0A089G3A2\|A0A089G3A2_CERNI Cytochrome c oxidase subunit 1 (Fragment) OS=Cervus nippon OX=9863 GN=COI PE=3 SV=1 | 11.9417 | 4247000 |  |
| TFH | 404.193 | 1 | 404.193 | 507 | 249.9 | >tr\|X2GM95\|X2GM95_CERNI Serum albumin (Fragment) OS=Cervus nippon OX=9863 PE=2 SV=1 | 22.1588 | 4207600 |  |
| WFP | 449.222 | 1 | 449.222 | 409 | 170.9 | >tr\|F1APT7\|F1APT7_CERNI Toll-like receptor 8 OS=Cervus nippon OX=9863 GN=TLR8 PE=2 SV=1 | 43.5105 | 4144300 |  |
| IGDF | 451.220 | 1 | 451.220 | 1151 | 185.2 | >tr\|F8V2T3\|F8V2T3_CERNI Tyrosine-protein kinase receptor OS=Cervus nippon OX=9863 PE=2 SV=1 | 26.2492 | 4074800 |  |
| VANAL | 487.289 | 1 | 487.289 | 136 | 248.8 | >tr\|A0A220IG97\|A0A220IG97_CERNI Adult beta-globin OS=Cervus nippon OX=9863 PE=3 SV=1 | 13.9947 | 4073500 |  |
| YVP | 378.203 | 1 | 378.203 | 496 | 164.8 | >tr\|X2GM95\|X2GM95_CERNI Serum albumin (Fragment) OS=Cervus nippon OX=9863 PE=2 SV=1 | 17.129 | 3997600 | ACE inhibitor |
| ECCHGDLLECADDR | 875.334 | 2 | 1749.661 | 243 | 252.1 | >tr\|X2GM95\|X2GM95_CERNI Serum albumin (Fragment) OS=Cervus nippon OX=9863 PE=2 SV=1 | 25.5137 | 3978300 |  |
| TDF | 382.161 | 1 | 382.161 | 47 | 202.9 | >tr\|X2GM95\|X2GM95_CERNI Serum albumin (Fragment) OS=Cervus nippon OX=9863 PE=2 SV=1 | 13.5181 | 3890500 |  |
| LET | 362.208 | 1 | 362.208 | 771 | 91.9 | >tr\|F8V2T3\|F8V2T3_CERNI Tyrosine-protein kinase receptor OS=Cervus nippon OX=9863 PE=2 SV=1 | 41.5089 | 3784400 |  |
| NFR | 436.233 | 1 | 436.233 | 101 | 109.8 | >tr\|A0A220IG97\|A0A220IG97_CERNI Adult beta-globin OS=Cervus nippon OX=9863 PE=3 SV=1 | 13.5918 | 3753000 |  |
| LTET | 463.225 | 1 | 463.225 | 592 | 93.1 | >tr\|F1APT7\|F1APT7_CERNI Toll-like receptor 8 OS=Cervus nippon OX=9863 GN=TLR8 PE=2 SV=1 | 46.9351 | 3728600 |  |
| HAFP | 471.226 | 1 | 471.226 | 86 | 58.0 | >tr\|A0A142EHU4\|A0A142EHU4_CERNI Matrix metallopeptidase (Fragment) OS=Cervus nippon OX=9863 GN=MMP-13 PE=2 SV=1 | 44.2983 | 3659200 |  |
| QTALVELLK | 507.815 | 2 | 1014.622 | 525 | 336.6 | >tr\|X2GM95\|X2GM95_CERNI Serum albumin (Fragment) OS=Cervus nippon OX=9863 PE=2 SV=1 | 46.552 | 3560400 |  |
| VGLP | 385.257 | 1 | 385.257 | 313 | 102.7 | >tr\|A4GIN2\|A4GIN2_CERNI Collagen alpha-1(X) chain OS=Cervus nippon OX=9863 GN=COL10A1 PE=2 SV=1 | 13.6464 | 3545400 |  |
| CCAKDDPHACY | 698.756 | 2 | 1396.505 | 359 | 102.2 | >tr\|X2GM95\|X2GM95_CERNI Serum albumin (Fragment) OS=Cervus nippon OX=9863 PE=2 SV=1 | 12.2491 | 3414200 |  |
| TPV | 316.187 | 1 | 316.187 | 122 | 210.4 | >tr\|A0A220IG97\|A0A220IG97_CERNI Adult beta-globin OS=Cervus nippon OX=9863 PE=3 SV=1 | 20.8638 | 3389600 |  |
| VGTKCCTKPE | 393.854 | 3 | 1179.549 | 432 | 160.2 | >tr\|X2GM95\|X2GM95_CERNI Serum albumin (Fragment) OS=Cervus nippon OX=9863 PE=2 SV=1 | 7.3185 | 3335900 |  |
| AHF | 374.171 | 1 | 374.171 | 62 | 48.8 | >tr\|G0Z3A2\|G0Z3A2_CERNI Catalase OS=Cervus nippon OX=9863 GN=CAT PE=2 SV=1 | 36.556 | 3308300 |  |
| SYGP | 423.190 | 1 | 423.190 | 9 | 113.5 | >tr\|Q2TQB3\|Q2TQB3_CERNI MHC class II antigen (Fragment) OS=Cervus nippon OX=9863 GN=Ceni-DQA1 PE=4 SV=1 | 18.3763 | 3285000 |  |
| YIV | 394.233 | 1 | 394.233 | 168 | 89.3 | >tr\|F8V2T3\|F8V2T3_CERNI Tyrosine-protein kinase receptor OS=Cervus nippon OX=9863 PE=2 SV=1 | 25.6677 | 3273000 |  |
| FTST | 455.202 | 1 | 455.202 | 495 | 40.3 | >tr\|F8V2T3\|F8V2T3_CERNI Tyrosine-protein kinase receptor OS=Cervus nippon OX=9863 PE=2 SV=1 | 10.3575 | 3241500 |  |
| EFM | 442.175 | 1 | 442.175 | 29 | 68.3 | >sp\|Q8WMR3\|GLHA_CERNI Glycoprotein hormones alpha chain OS=Cervus nippon OX=9863 GN=CGA PE=2 SV=1 | 45.8795 | 3154100 |  |
| TMR | 407.205 | 1 | 407.205 | 183 | 18.8 | >tr\|X2GM95\|X2GM95_CERNI Serum albumin (Fragment) OS=Cervus nippon OX=9863 PE=2 SV=1 | 56.5226 | 3149000 |  |
| KPDV | 458.264 | 1 | 458.264 | 23 | 42.2 | >tr\|G0Z3A2\|G0Z3A2_CERNI Catalase OS=Cervus nippon OX=9863 GN=CAT PE=2 SV=1 | 14.7572 | 2999100 |  |
| SYL | 382.187 | 1 | 382.187 | 655 | 125.9 | >tr\|F8V2T3\|F8V2T3_CERNI Tyrosine-protein kinase receptor OS=Cervus nippon OX=9863 PE=2 SV=1 | 23.3051 | 2908200 |  |
| AYF | 400.189 | 1 | 400.189 | 301 | 62.8 | >tr\|G9F9N5\|G9F9N5_CERNI Melatonin receptor OS=Cervus nippon OX=9863 GN=MTNR1A PE=3 SV=1 | 42.6606 | 2712300 |  |
| TTW | 407.204 | 1 | 407.204 | 499 | 55.8 | >tr\|F8V2T3\|F8V2T3_CERNI Tyrosine-protein kinase receptor OS=Cervus nippon OX=9863 PE=2 SV=1 | 54.1493 | 2553300 | ACE inhibitor |
| TMP | 348.161 | 1 | 348.161 | 825 | 54.8 | >tr\|F8V2T3\|F8V2T3_CERNI Tyrosine-protein kinase receptor OS=Cervus nippon OX=9863 PE=2 SV=1 | 9.6616 | 2523300 |  |
| TVFD | 481.232 | 1 | 481.232 | 371 | 98.5 | >tr\|X2GM95\|X2GM95_CERNI Serum albumin (Fragment) OS=Cervus nippon OX=9863 PE=2 SV=1 | 23.7492 | 2470800 |  |
| MIGAP | 488.252 | 1 | 488.252 | 71 | 95.4 | >tr\|A0A089G3A2\|A0A089G3A2_CERNI Cytochrome c oxidase subunit 1 (Fragment) OS=Cervus nippon OX=9863 GN=COI PE=3 SV=1 | 32.5922 | 2452700 |  |
| LVVYPWTQ | 1005.546 | 1 | 1005.546 | 31 | 113.4 | >tr\|A0A220IG97\|A0A220IG97_CERNI Adult beta-globin OS=Cervus nippon OX=9863 PE=3 SV=1 | 53.0636 | 2319800 | opioid /ACE inhibitor |
| TMLV | 463.257 | 1 | 463.257 | 74 | 51.0 | >sp\|Q8WMR3\|GLHA_CERNI Glycoprotein hormones alpha chain OS=Cervus nippon OX=9863 GN=CGA PE=2 SV=1 | 27.8678 | 2197400 |  |
| ECCDKPVL | 510.730 | 2 | 1020.454 | 276 | 143.8 | >tr\|X2GM95\|X2GM95_CERNI Serum albumin (Fragment) OS=Cervus nippon OX=9863 PE=2 SV=1 | 18.5639 | 2167300 |  |
| SIF | 366.203 | 1 | 366.203 | 847 | 87.4 | >tr\|F8V2T3\|F8V2T3_CERNI Tyrosine-protein kinase receptor OS=Cervus nippon OX=9863 PE=2 SV=1 | 31.6975 | 1882400 |  |
| TVVAP | 486.292 | 1 | 486.292 | 37 | 203.7 | >tr\|D3YJ54\|D3YJ54_CERNI Insulin-like growth factor II OS=Cervus nippon OX=9863 GN=IGF2 PE=2 SV=1 | 29.2204 | 1869200 |  |
| IPVG | 385.245 | 1 | 385.245 | 311 | 32.6 | >tr\|G0Z3A2\|G0Z3A2_CERNI Catalase OS=Cervus nippon OX=9863 GN=CAT PE=2 SV=1 | 24.3142 | 1837400 |  |
| SVPGK | 487.298 | 1 | 487.298 | 153 | 22.7 | >tr\|A4GIN2\|A4GIN2_CERNI Collagen alpha-1(X) chain OS=Cervus nippon OX=9863 GN=COL10A1 PE=2 SV=1 | 31.9981 | 1829600 |  |
| EAT | 320.160 | 1 | 320.160 | 353 | 47.8 | >tr\|X2GM95\|X2GM95_CERNI Serum albumin (Fragment) OS=Cervus nippon OX=9863 PE=2 SV=1 | 17.7154 | 1758700 |  |
| AFWG | 480.227 | 1 | 480.227 | 12 | 110.8 | >tr\|A0A220IG97\|A0A220IG97_CERNI Adult beta-globin OS=Cervus nippon OX=9863 PE=3 SV=1 | 38.7733 | 1705800 |  |
| KGV | 303.202 | 1 | 303.202 | 1019 | 154.4 | >tr\|F8V2T3\|F8V2T3_CERNI Tyrosine-protein kinase receptor OS=Cervus nippon OX=9863 PE=2 SV=1 | 14.4269 | 1705600 |  |
| SSSQ | 408.181 | 1 | 408.181 | 623 | 12.5 | >tr\|F8V2T3\|F8V2T3_CERNI Tyrosine-protein kinase receptor OS=Cervus nippon OX=9863 PE=2 SV=1 | 43.1939 | 1677600 |  |
| LTEGDHGFH | 506.729 | 2 | 1012.450 | 36 | 208.0 | >tr\|V5LTF3\|V5LTF3_CERNI Cu/Zn superoxide dismutase (Fragment) OS=Cervus nippon OX=9863 PE=2 SV=1 | 22.6184 | 1666200 |  |
| SLR | 375.235 | 1 | 375.235 | 79 | 100.4 | >tr\|X2GM95\|X2GM95_CERNI Serum albumin (Fragment) OS=Cervus nippon OX=9863 PE=2 SV=1 | 10.9878 | 1661600 | ACE inhibitor |
| VFL | 378.240 | 1 | 378.240 | 324 | 149.2 | >tr\|X2GM95\|X2GM95_CERNI Serum albumin (Fragment) OS=Cervus nippon OX=9863 PE=2 SV=1 | 38.7271 | 1630800 |  |
| VDKCCAAVDK | 583.272 | 2 | 1165.537 | 554 | 175.8 | >tr\|X2GM95\|X2GM95_CERNI Serum albumin (Fragment) OS=Cervus nippon OX=9863 PE=2 SV=1 | 8.3766 | 1619000 |  |
| LLPVRWMSP | 557.798 | 2 | 1114.589 | 1173 | 111.1 | >tr\|F8V2T3\|F8V2T3_CERNI Tyrosine-protein kinase receptor OS=Cervus nippon OX=9863 PE=2 SV=1 | 49.975 | 1371900 |  |
| VGDL | 403.233 | 1 | 403.233 | 79 | 19.9 | >tr\|V5LTF3\|V5LTF3_CERNI Cu/Zn superoxide dismutase (Fragment) OS=Cervus nippon OX=9863 PE=2 SV=1 | 11.8028 | 1128700 |  |
| AHGP | 381.175 | 1 | 381.175 | 9 | 9.1 | >tr\|Q2TQA0\|Q2TQA0_CERNI MHC class II antigen (Fragment) OS=Cervus nippon OX=9863 GN=Ceni-DQA2 PE=4 SV=1 | 8.7387 | 731620 |  |
| PFP | 360.191 | 1 | 360.191 | 257 | 156.0 | >tr\|F1APT7\|F1APT7_CERNI Toll-like receptor 8 OS=Cervus nippon OX=9863 GN=TLR8 PE=2 SV=1 | 32.6256 | 716900 | ACE inhibitor |
| IDIH | 497.285 | 1 | 497.285 | 801 | 12.5 | >tr\|F8V2T3\|F8V2T3_CERNI Tyrosine-protein kinase receptor OS=Cervus nippon OX=9863 PE=2 SV=1 | 9.3228 | 706490 |  |
| VLY | 394.233 | 1 | 394.233 | 971 | 63.6 | >tr\|F8V2T3\|F8V2T3_CERNI Tyrosine-protein kinase receptor OS=Cervus nippon OX=9863 PE=2 SV=1 | 47.6947 | 602870 | ACE inhibitor |
| KPPK | 469.315 | 1 | 469.315 | 173 | 116.3 | >tr\|F8V2T3\|F8V2T3_CERNI Tyrosine-protein kinase receptor OS=Cervus nippon OX=9863 PE=2 SV=1 | 8.6699 | 434850 |  |
| AGVTH | 484.263 | 1 | 484.263 | 564 | 30.4 | >tr\|F1APT7\|F1APT7_CERNI Toll-like receptor 8 OS=Cervus nippon OX=9863 GN=TLR8 PE=2 SV=1 | 43.0003 | 352860 |  |
